# Decreased *ZNF750* promotes angiogenesis in a paracrine manner via activating *DANCR/miR-4707-3p/FOXC2* axis in esophageal squamous cell carcinoma

**DOI:** 10.1038/s41419-020-2492-2

**Published:** 2020-04-27

**Authors:** Yanghui Bi, Shixing Guo, Xiaoqin Xu, Pengzhou Kong, Heyang Cui, Ting Yan, Yanchun Ma, Yikun Cheng, Yunqing Chen, Xue Liu, Ling Zhang, Caixia Cheng, Enwei Xu, Yu Qian, Jian Yang, Bin Song, Hongyi Li, Fang Wang, Xiaoling Hu, Xiangchen Liu, Xia Niu, Yuanfang Zhai, Jing Liu, Yaoping Li, Xiaolong Cheng, Yongping Cui

**Affiliations:** 1 Department of Pathology & Shanxi Key Laboratory of Carcinogenesis and Translational Research of Esophageal Cancer, Shanxi Medical University, Taiyuan, Shanxi 030001 P.R. China; 2 Shenzhen Peking University-Hong Kong University of Science and Technology (PKU-HKUST) Medical Center, Peking University Shenzhen Hospital, Shenzhen, 518035 P.R. China; 3 College of Letter & Science, University of California Berkeley, Berkeley, California, 94704 USA; 4 Surgery Special Wards, Shanxi Cancer Hospital, Taiyuan, Shanxi 030001 P.R. China; 5 Department of Pathology, the First Hospital, Shanxi Medical University, Taiyuan, Shanxi 030001 P.R. China; 6 Department of Pathology, Shanxi Cancer Hospital, Taiyuan, Shanxi 030001 P.R. China; 7 Department of Oncology, The First Hospital, Shanxi Medical University, Taiyuan, Shanxi 030001 P.R. China; 8 Department of General Surgery, The First Hospital, Shanxi Medical University, Taiyuan, Shanxi 030001 P.R. China; 9 Department of Colorectal & Anal Surgery, Affiliated Provincial Hospital of Shanxi Medical University, Taiyuan, Shanxi 030001 P.R. China

**Keywords:** Oesophageal cancer, Mechanisms of disease

## Abstract

*ZNF750* is one novel significantly mutated gene identified in esophageal squamous cell carcinoma (ESCC) using next-generation sequencing. However, its clinically relevant and potential mechanisms have remained elusive. Using genomic sequencing of 612 ESCC patients, we analyzed the associations of *ZNF750* mutations with clinicopathologic features and its prognostic value. We further investigated the function and underlying mechanism of *ZNF750* in angiogenesis. The results showed *ZNF750* mutations/deletions are significantly associated with malignant progression and poor prognosis of ESCC patients. Decreased *ZNF750* in ESCC cells induces enhanced angiogenesis of human umbilical vein endothelial cells (HUVECs) and human arterial endothelial cells (HAECs), and the effect may be indirectly mediated by FOXC2. RNA-seq and ChIP shows lncRNA *DANCR* is a direct downstream target of *ZNF750*. Furtherly, knockdown *ZNF750* evokes *DANCR* expression, which prevents *miR-4707-3p* to interact with *FOXC2* as a microRNA sponge in a ceRNA manner, leading to enhanced FOXC2 signaling and angiogenesis. In contrast, *ZNF750* expression reverses the effect. Our study reveals a novel mechanism of *ZNF750*, highlights a significance of *ZNF750* as a metastatic and prognostic biomarker, and offers potential therapeutic targets for ESCC patients harboring *ZNF750* mutations.

## Introduction

Esophageal squamous cell carcinoma (ESCC), the major type of esophageal cancers in China accounting for over 477,900 new cases and 375,000 deaths recorded annually, remains one of the most lethal of malignancies and a major health burden^1^. Although chemotherapy and radiotherapy can improve the disease outcome, relapse is frequent and treatment options and molecular markers are limited, leading to a poor prognosis with 20% of 5-year survival rates in China^1–3^.

Recent studies have profiled ESCC genomic alterations and have identified significantly mutated genes (SMGs) including *TP53*, *ZNF750*, *NOTCH1*, *FAT1*, *NFE2L2*, copy number amplifications occurring in *SOX2*, *TERT*, *FGFR1*, *MDM1*, and common deletions of *RB1* etc^4–7^. Of these genes, *ZNF750*, a nuclear factor that plays a critical role in control of terminal epidermal differentiation gene program^8,9^, was frequently disrupted by somatic inactivating mutations on 17q25.3^4,6^. ZNF750 has been reported to directly regulate FGF14 promoting cell apoptosis thus inhibited nasopharyngeal carcinoma^10^. Meanwhile, ZNF750 inhibited the malignant progression of oral squamous cell carcinoma by regulating tumor vascular microenvironment^11^. TINCR lncRNA, one of the downstream targets of ZNF750, participated in control of cellular proliferation, migration, and differentiation of SCC cells. ZNF750 also suppressed migration of SCC cells by directly inhibiting transactivation of LAMC2^12^. Recent attempts to decipher the significance of *ZNF750* for tumorigenesis in ESCC have revealed that *ZNF750* might act as a tumor suppressor gene^4,6^. However, the molecular mechanism underlying *ZNF750* contributes to tumorigenesis and the clinically relevant of genetic changes of *ZNF750* in ESCC remain largely unresolved.

In this study, we reveal the associations *ZNF750* mutations and/or deletions with clinical variables using genomic sequencing data of 612 pairs of ESCC tumor and normal samples from China and explore the molecular mechanism through which *ZNF750* plays a critical role in driving the formation of metastatic ESCC. We found loss-function of *ZNF750* evokes *DANCR* expression and prevents *miR-4707-3p* to interact with *FOXC2* mRNA in a ceRNA manner, leading to enhanced FOXC2 signaling and angiogenesis phenotype in ESCC. Importantly, we performed RNA-sequencing of paired fresh tumor tissues and matched adjacent non-cancerous specimens from 97 ESCC subjects; together with available TCGA data, we validated the associations among *ZNF750* and the identified downstream targets in ESCC and other squamous carcinomas. Our findings reveal an underlying mechanism by which loss-function of *ZNF750* contributes to ESCC progression, provide a potential metastatic and prognostic biomarker and several therapeutic targets for ESCC patients harboring *ZNF750* mutations and/or deletions.

## Materials and methods

### Samples and clinical data

Tumor samples and adjacent normal tissues with good quality and sufficient quantity for in-depth pathological and molecular investigation were obtained from 508 ESCC patients recruited from Shanxi and Xinjiang provinces, China. All patients have given their informed consent and all samples were obtained before treatment according to the guidelines of the Shanxi Medical University Medical ethical committees. The 508 pairs of tumor and normal samples were subjected to hematoxylin and eosin (HE) staining. The stained sections from each sample were reviewed by at least three independent pathologists to confirm that the tumor specimen was histologically consistent with ESCC and the adjacent tissue specimen contained no tumor cells. Together with our previous 104 ESCC patients recruited from the Taihang Mountain of North-Central China, we analyzed the associations of *ZNF750* mutations with patients’ clinical features in a cohort of 612 ESCC patients in this study. Medical records and survival data were obtained for all 612 of ESCC patients. The clinical, epidemiological or pathological features were showed in Table 1.

**Table 1:** Correlation analysis between *ZNF750* genotypes in 612 ESCC samples and clinicopathological variables.

Clinical, epidemiological or pathological feature	*ZNF750_mut*	Proportion	*ZNF750_wt*	Proportion	*P value*	*ZNF750_mut*
All cases	612	48	7.84%	564	92.16%	
Age						
<60	270	23	8.52%	247	91.48%	
60–69	264	17	6.44%	247	93.56%	0.457
≥70	78	8	10.26%	70	89.74%	
Sex						
Male	439	39	8.88%	400	91.12%	0.137
Female	173	9	5.20%	164	94.80%	
Tobacco use						
No	262	17	6.49%	245	93.51%	0.293
Yes	350	31	8.86%	319	91.14%	
Alcohol consumption						
No	391	27	6.91%	364	93.09%	0.274
Yes	221	21	9.50%	200	90.50%	
Tumor location						
Upper thoracic	198	3	1.52%	195	98.48%	
Middle thoracic	364	22	6.04%	342	93.96%	3.1427E-16
Lower thoracic	50	23	46.00%	27	54.00%	
Histological grade						
Grade 1	71	3	4.23%	68	95.77%	0.145
Grade 2	402	29	7.21%	373	92.79%	0.145
Grade 3	139	16	11.51%	123	88.49%	
Pathologic Stage						
I&II	374	16	4.28%	358	95.72%	8E-05
III&IV	238	32	13.45%	206	86.55%	
Pathologic T Stage						
T1	47	4	8.51%	43	91.49%	
T2	213	13	6.10%	200	93.90%	0.003
T3	341	26	7.62%	315	92.38%	
T4	11	5	45.45%	6	54.55%	
Pathologic N Stage						
N0	355	16	4.51%	339	95.49%	
N1	147	15	10.20%	132	89.80%	0.001
N2	79	13	16.46%	66	83.54%	
N3	31	4	12.90%	27	87.10%	
Lymphatic metastasis						
Yes	257	33	12.84%	224	87.16%	0.0001
No	355	15	4.23%	340	95.77%	
Prognosis (Log-rank Mantel-Cox test)						
Dead	263	29	11.03%	234	88.97%	
Survival	324	17	5.25%	307	94.75%	0.026
Missing	25	2	8.00%	23	92.00%	

### DNA extraction and whole-genome sequencing

After microdissection procedure, an average tumor content of 55% was achieved. We also imputed deviation in the allele frequency of heterozygous single-nucleotide variation to predict the tumor purity and ploidy for each sample with a median of 51.18%. Genomic DNA was extracted by Maxwell 16 Tissue DNA Purification Kit (Promega) according to the manufacturer’s instructions. DNA integrity and concentration were determined by Qubit 2.0 fluorometer dsDNA HS Assay (Thermo Fisher Scientific) and NanoDrop2000 (Thermo Fisher Scientific). Fragmented DNA (~350 bp) was purified using Sample Purification Beads (Illumina). Adapter-ligated libraries were prepared with the TruSeq Nano DNA Sample Prep Kits (Illumina) according to Illumina’s protocol. Illumina cBOT cluster generation system with HiSeq PE Cluster Kits (Illumine) was used to generate clusters. Paired-end whole-genome sequencing (WGS) was performed using an Illumina HiSeq system following Illumina’s instructions for 2 × 150 paired-end sequencing in WuXi NextCODE at Shanghai, China.

### *ZNF750* genetic variants calling

We used previously reported common method and algorithms to detect somatic mutation variations. High-quality reads were aligned to the UCSC human reference genome (hg19) using Burrows-Wheeler Aligner (BWA v.0.7.12) with default parameters. Variants calling was performed using Sentieon algorithm (https://www.sentieon.com/). For each paired sample, somatic single-nucleotide variants (SNVs) and small insertions and deletions (indels) were detected by MuTect2 (http://archive.broadinstitute.org/cancer/cga/mutect). Significantly mutated genes (SMGs) were identified using the MutSigCV tools (http://archive.broadinstitute.org/cancer/cga/mutsig); q (FDR) < 0.001 was considered significantly mutated. We used the software CNVkit (v0.8.3) to analyze sequencing coverage and copy number in the aligned sequencing reads.

For detection of DNA copy number variation, we performed SegSeq to infer somatic copy number variation (CNV) in ESCC genomes based on WGS reads. Copy numbers of ≤1.5 were considered to indicate deletions and ≥2.5 were considered as amplifications. To infer recurrently amplified or deleted genomic regions, we re-implemented GISTIC algorithm using copy numbers in 1-kb windows as markers instead of SNP array probes. G-scores were calculated for genomic and gene-coding regions based on the frequency and amplitude of amplification or deletion affecting each gene. A significant CNV region was defined as having amplification or deletion with G-score > 0.1, corresponding to a *p*-value threshold of 0.05 from permutation-derived null distribution.

### qRT-PCR copy number analysis

*ZNF750* copy number was assessed in frozen seven tumor samples and matched normal tissues. Copy numbers were determined by real-time PCR with DNA binding dye SYBR Green I using three highly specific primer pairs that flanked three coding exons of each gene. In a final volume of 25 µl, 20 ng DNA was amplified with SYBR Green PCR Master Mix (QIAGEN, Germany) in triplicate. RNase P (*RPPH1* gene; Life Technologies, 4403328) was used as a diploid control and *TMC8* (chr17:76125505-76139049) was used as control located in the region nearby *ZNF750* gene. Data was analyzed using the comparative (delta-Ct) Ct method. The primers are listed in Table S1.

### Cell lines

Immortalized esophageal epithelial cell SHEE and ESCC cell lines KYSE140, KYSE150, KYSE180, KYSE410, KYSE510, KYSE450, Colo680N, ECA109 were stored at Shanxi key laboratory of carcinogenesis and translational research on esophageal cancer, Shanxi Medical University. Cells were grown in RPMI1640 media supplementary with 10% fetal bovine serum (FBS), 100 U/ml penicillin, and 100 μg/ml streptomycin. Human umbilical vein endothelial cell (HUVEC) line EA.hy926 was purchased from ATCC and cultured in DMEM-low glucose (Hyclone, USA) with 10% FBS, 100 U/ml penicillin, and 100 μg/ml streptomycin. Human arterial endothelial cell (HAECs) line was stored at Shanxi key laboratory of carcinogenesis and translational research on esophageal cancer, Shanxi Medical University and cultured in Endothelial Cell Medium (ScienCell, USA), with 5% FBS, 1% Endothelial Cell Growth Supplement (ECGS), and 1% penicillin/streptomycin. All cell lines were identified by STR and no mycoplasma contamination.

### Plasmids construction and transfections

ZNF750 expression plasmid, a generous gift from Paul A. Khavari professor (Stanford University), was sub-cloned into the pcDNA3.1 vector with a HA tag and validated by sequencing and Western blot. The luciferase reporter plasmid of *DANCR* promoter with mutant or deleted ZNF750 binding site was constructed using pGL3-basic vector. The luciferase reporter plasmids of *DANCR-wt*, *DANCR-mut*, *FOXC2-wt*, *FOXC2-mut* were constructed using pSicheck2. The siRNA (RiboBio, Guangzhou, China) specific for *DANCR* or *FOXC2* were used to knockdown DANCR or FOXC2, respectively. Transfections were performed using lipofectamie 2000 (Invitrogen, USA) following the manufacturer’s instructions. The siRNA for target genes, *miR-4707-3p* mimics and miRNA inhibitors were purchased from RiboBio (RiboBio, China). The lentivirus for stable expression or knockdown were constructed and packaged by GenePharma Co. (Shanghai, China). The siRNA sequences targeting specific genes are shown in Supplementary Table S1. Plasmids transfection was performed *via* the lipofectamine^TM^ 2000 transfection reagent (Invitrogen) according to the manufacturer.

### Conditioned media preparation

Conditioned media (CM) preparation was performed as described previously. Briefly, ESCC cells with *ZNF750* overexpression and knockdown were grown on 100 mm plates in about 70–80% confluence and then transformed into serum free media for 24 h. Then media were collected and centrifugated at 2000 rpm at the temperature of 4 °C for 10 min. After being centrifuged at 12,000 rpm for 20 min, the supernatants were filtered through 0.22 μm sterile filter and stored at 4 °C. Concentrated media were normalized of the cell number of different groups. When used, all CM were diluted 1:1 with the corresponding fresh DMEM complete medium.

### MTT assay

In order to examine the effect of ZNF750 on HUVEC and HAEC cell viability, cells were digested to prepare single-cell suspensions and then seeded at a density of 5×10^3^/well into 96-well plates with conditioned media. At different time points, 20 µl of 5 mg/ml MTT (Invitrogen, USA) was added into each well and incubated for 4 h at 37 °C. Then MTT solution was removed from each well and 150 μl of DMSO was added to dissolve the crystals. Color intensity was measured by an ELISA reader at 490 nm. Each experiment consisted of three replications and at least three independent experiments were carried out.

### Migration and invasion assays

To examine the effect of ZNF750 on the migration and invasion ability of HUVEC and HAEC cells, transwell migration/invasion assays were performed in the Biocoat Matrigel Invasion Chamber (BD Biosciences) according to the manufacturer’s instructions. Briefly, 2×10^4^ cells were plated into the upper chambers of a 24-well plate and cultured with FBS free medium. The bottom chambers contained 600 μl of CM. After 24 h, cells on the upper surface were removed, fixed with 4% paraformaldehyde, and stained with 0.1% crystal violet. Microscopy (Olympus, Japan) was used to image the cells that transmigrated to the underneath surface of transwell membrane. Randomly selected four fields of transmigrated cells and manually counted. For the transwell invasion assay, the upper chambers consisting of 8 μm membrane filter inserts coated with Matrigel (BD Biosciences). Each experiment consisted of three replications and at least three independent experiments were carried out.

### Western blot

Total proteins were extracted using RIPA buffer (Sigma, USA) containing protease and phosphatase inhibitors (Thermo Fisher Scientific) on ice for one hour, and the protein concentration was detected by a BCA assay kit (Real-Times (Beijing) Biotechnology Co., Ltd., Beijing, China). After being separated on a 10% SDS-polyacrylamide gel, the proteins were transferred onto polyvinylidene fluoride (PVDF) membranes (Whatman GmbH, Maidstone, Kent, UK) that were subjected to blocking by 5% skimmed milk for two hours at room temperature, then incubated with specific antibodies at 4 °C overnight. The blot was detected with horseradish peroxidase labeled secondary antibody (Sigma, USA, 11520709001), and chemiluminescence was detected with a LAS4000 device (Fuji). ZNF750 or FOXC2 protein levels in ESCCs were determined by western blot with ZNF750 antibody (Santa, USA, sc-292024) or FOXC2 antibody (Proteintech, USA, 23066-1-AP). β-actin (Proteintech, USA, 66009-1-Ig) was used as loading control.

### Vasculogenic mimicry (Tube formation assay)

Tube formation assay was performed to exam the effect of ZNF750 on angiogenesis ability of HUVEC and HAEC cells. Briefly, 50 μl Matrigel (BD Biosciences, Bedford, Massachusetts, USA) was added into each well of a 96-well plate for 30 min, 37 °C. Then cells (3 × 10^5^) in 50 μl of conditioned medium were added to each well and incubated at 37 °C, 5% CO_2_ for 8 h. Images were taken using a bright-field microscope at ×40 magnification. We randomly selected 10 views and captured the formation of tube-like structures under an inverted light microscope. The capillary tubes were quantified by measuring the total numbers of completed tubule structures. The number and length of tubes were calculated with Image J software.

### Mouse xenograft assay and immunohistochemistry (IHC)

To further determine the effects of *DANCR* on tumorigenesis in vivo, we used a mouse xenograft assay with 4–6-week-old BALB/c nude female mice (Vital River Laboratory Animal Technology Co., Ltd., Beijing, China). Randomization was conducted. 5 × 10^6^ cells were subcutaneously injected into the left or right oxter of nude mice (*n* = 5 mice/group). The growth rates of xenograft tumors were measured every four days. After 4 weeks, tumors were removed, snap frozen in liquid nitrogen, and stored at −80 °C. Tumor size was measured with calipers. Additionally, tissue micro-array production as follows, we put 508 ESCC tumors and corresponding non-tumor donor tissues recruited from Shanxi and Xinjiang provinces into corresponding holes of the blank receptor wax block and repeated freezing and thawing the new wax block to make them together. FFPE xenograft tumors and ESCC tissue microarrays were immunohistochemically stained with specific antibodies as described previously^4^. The antibodies used in this experiment are shown as follows: Ki-67 (Zhongshan, China, ZM-0166), ZNF750 (Sigma, USA, HPA021573), FOXC2 (Proteintech, USA, 23066-1-AP), CD31(Abcam, USA, ab134168), FLT1 (Proteintech, USA, 13687-1-AP), ANGPT2 (Proteintech, USA, 24613-1-AP). All images were captured at 200× magnification. The nuclear amount of proteins was analyzed with Aperio Nuclear v.9 software. Statistical analyses were performed with GraphPad Prism 7.0. All animal study in this study was approved by the Experimental Animal Welfare Ethics Committee of Shanxi Medical University.

### RNA-sequencing

We performed RNA-sequencing on fresh tumor specimens and matched adjacent normal tissues from 97 ESCC patients recruited from Shanxi province, China. We also applied RNA-sequencing to KYSE150 cells with *ZNF750-wt* overexpression. Total RNAs were isolated from tissue samples or cell lines using the TRIzol reagent (Life Technologies, Carlsbad, CA, USA) and DNA was digested by DNase I according to manufacturer’s protocols. RNA quantity and quality were evaluated by NanoDrop spectrophotometer (Thermo Scientifc, USA). mRNA was isolated from total RNA using the oligo‐dT magnetic beads and braked into fragments for cDNA libraries construction. The cDNA libraries were quality inspection qualified with the Agilent 2100 Bioanalyzer and ABI Step One Plus Real-Time PCR System, then sequenced on WuXi NextCODE Genomics (Shanghai) Co., Ltd., China. Raw reads were subjected to quality control (QC), filtered into clean reads, then aligned to the reference sequences. The alignment data were utilized to calculate distribution of reads on reference genes and mapping ratio. If alignment result passes QC, we proceeded the analyses including gene and isoform expression, deep analysis based on gene expression (PCA/correlation/screening differently expressed genes and so on), exon expression, gene structure refinement, alternative splicing, novel transcript prediction and annotation, SNV detection, indel detection. Based on differentially expressed genes (DEGs), we also performed deep analyses including gene ontology (GO) enrichment, pathway enrichment, cluster, protein-protein interaction network, and identification of transcription factor. The TargetScan (targetscan.org) and miRTarBase (mirtarbase.mbc.nctu.edu.tw) software packages were used to predict the targets of miRNAs.

### Chromatin immunoprecipitation (ChIP)

ChIP was performed using a Chip assay kit as recommended by the manufacturer (Millipore, USA). Approximately 1 × 10^7^ cells transfected with pcDNA3.1-HA *ZNF750-wt* were cross-linked in 1% formaldehyde at 37 °C for 10 min, and then 1.25 M glycine was added to quench the excess formaldehyde at room temperature for 5 min. Cells were washed twice in ice-cold PBS containing protease inhibitors, lysed in 200 μl of SDS lysis buffer for 10 min on ice, sonicated to break DNA into fragments of less than 1 kb and centrifuged at 12,000*g* for 15 min at 4 °C. The sonicated cell supernatants were subjected to immunoprecipitation (IP) with the following antibodies respectively, anti-HA (Abcam, USA, ab9110) or normal IgG (proteintech, USA, B900610). 1 mg of antibodies was used for each IP. After washing, immunoprecipitated DNA was eluted with elution buffer containing 1% SDS and 0.1 M NaHCO_3_, and the cross-links were reversed by incubation at 65 °C for 4 h in the presence of 200 mM NaCl and RNase A. After deproteinization with proteinase K, DNA was recovered by phenol-chloroform extraction and ethanol precipitation. Ordinarily PCR was performed to amplify the target DNA fragments which were visualized by agarose gel electrophoresis. The primers are listed in Table S1.

### Dual luciferase reporter assay

The luciferase activities were measured using a luciferase reporter assay system according to the manufacturer’s instructions (Transgene, Beijing, China). Briefly, cells were washed with ice-cold PBS and harvested in reporter lysis buffer. After centrifugation, 10 μl of the supernatants were mixed with 50 μl of solution1 and measured for firefly luciferase activity by using a TransDetect double-luciferase reporter assay kit (Transgene, China). Then mixed with 50 μl solution 2 and measured for Renilla luciferase activity.

### RNA-binding protein immunoprecipitation (RIP)

RIP was performed to assess the interaction between *DANCR* and *miR-4707-3p* using a Magna RIP kit (Millipore, USA) according to the manufacturer’s instruction. Briefly, cells were lysed in RIP lysis buffer, and suitable proportion was stored as input. Cell lysate was incubated with RIP buffer containing magnetic beads conjugated with human anti-Ago2 antibody (Sigma, USA, HPA058075) or anti-IgG (proteintech, USA, 10283-1-AP), the negative control, at room temperature for 30 min, then washed twice with wash buffer. Cell lysates were incubated with the magnetic beads overnight at 4 °C, then the beads were collected and washed several times. During all the procedures, RNase inhibitor cocktail was added to prevent RNA degradation. RNA on the beads was extracted by the phenol-chloroform method according to the instruction and analyzed by qRT-PCR to demonstrate the presence of the binding targets.

### Fluorescent in situ hybridization (FISH)

*DANCR* FISH staining was performed according to the manufacturer’s instruction. Briefly, cells were washed with 1× PBS for 5 min, fixed with 4% paraformldehyde at room temperature for 10 min, washed and permeabilization in 1 ml ice-cold 1×PBS with 0.5% Triton X-100, then subjected to pre-hybridization solution blocking the samples at 37 °C for 30 min. The 5 μM of anti-DANCR probe and anti-18s probe (RiboBio, China) were incubated in 100 μl hybridization solution at 37 °C overnight in a dark place. Samples were then washed three times for 15 min (5 min/each time) at 42 °C in a 4 × SSC solution with 0.1% Tween-20, once for 5 min in 2 × SSC, once for 5 min in 1 × SSC in a dark place, and once for 5 min at room temperature in PBS. DAPI in 50% glycerol was applied before imaging.

### Statistical analysis

Sample size was chosen based on the need for statistical power. All statistical evaluations were performed using the SPSS 22 software package (IBS SPSS, Armonk, NY, USA) and GraphPad Prism 6 (GraphPad Software, San Diego, California, USA). Rank sum and Chi square (χ^2^) tests were used to analyze the association of *ZNF750* mutations with clinical and pathological features. The follow-up period for progression-free survival was defined as the interval between the date of diagnosis of metastatic disease and that of progression disease. Survival curves were constructed using the Kaplan-Meier method and differences in survival were evaluated using the log-rank test. Overall survival (OS) was evaluated from the time of diagnosis to death or the final follow-up. Censored cases were defined as patients who lost contact during the follow-up and who were still alive at the end of the study. Univariate and multivariate survival analyses were performed by a Cox proportional hazards regression model.

All experiments were done in triplicates and data were presented as mean ± SD. Student’s *t* test was used for statistical analysis, and data from more than two groups were analyzed by one-way analysis of variance (ANOVA) followed by Dunnett’s test. The variance between the groups that are being statistically compared is similar. The correlation between *ZNF750* and *FOXC2* expression level was analyzed using nonparametric correlation (Spearman) in GraphPad Prism 6. A two-side *P* value < 0.05 was considered statistically significant.

## Results

### Recurrent losses of *ZNF750* is associated with metastasis and poor prognosis in ESCC patients

**Fig. 1:**
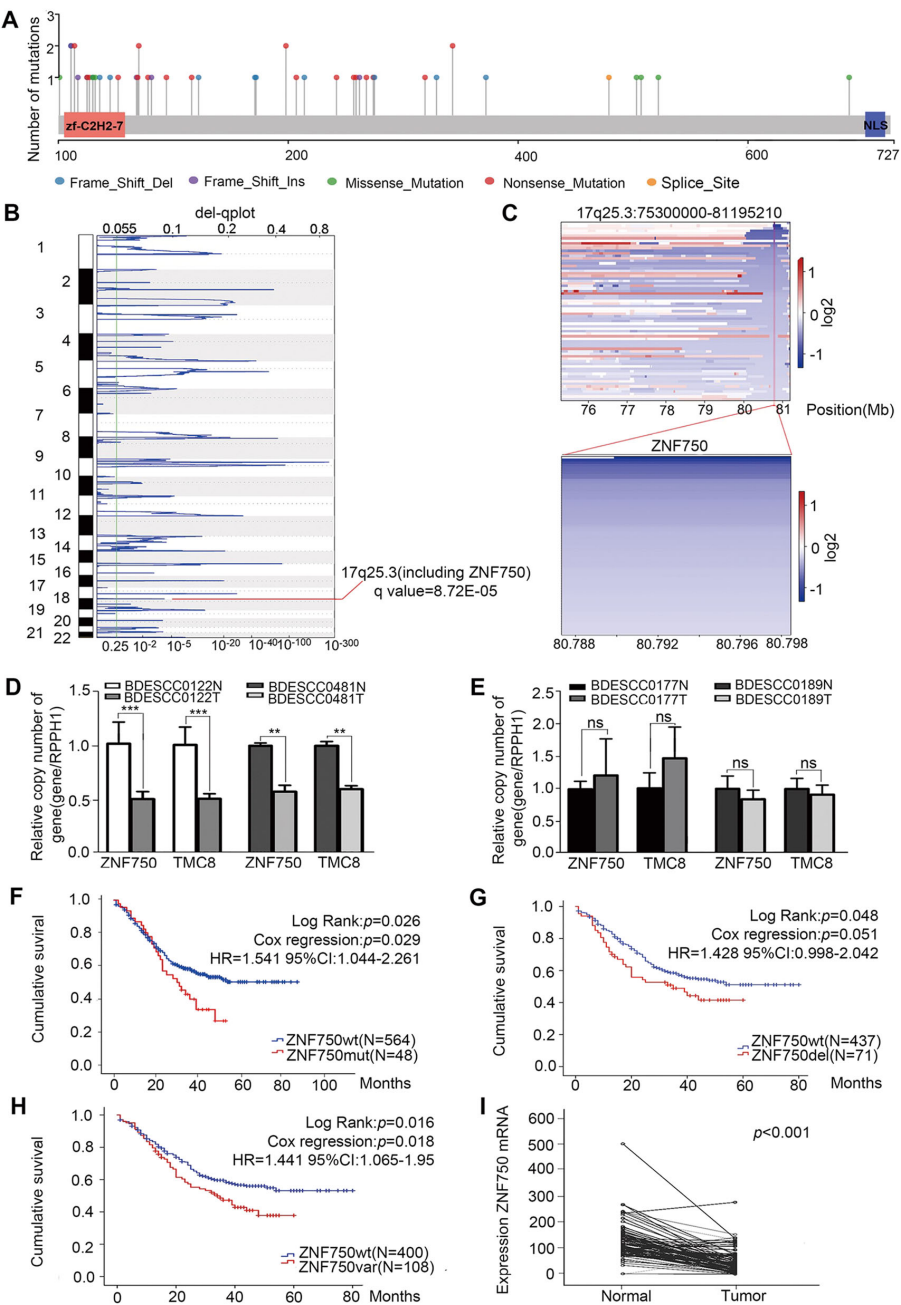
Genetic alterations of *ZNF750* across 612 ESCC genomes. **a** The mutation sites and types in CDS region of *ZNF750* across 612 ESCC genomes. **b** The significant focal SCNA filtered by GISTCI across 508 ESCC genomes. **c** Heatmap of CNV log2 ratio of read coverage across 71 ESCC individuals in 17q25.3 and *ZNF750* regions (upper) and detected significant deletion of *ZNF750* (bottom). **d**, **e** *ZNF750* copy number was assayed by qRT-PCR in BDESCC0122, BDESCC0481, BDESCC0177 and BDESCC0189. *RPPH1* was used as a normal reference, and *TMC8* (located near this region) was used as a focal CNA control. **f**–**h** Kaplan–Meier survival analysis between ESCC patients with *ZNF750-mut* and *ZNF750-wt*, ESCC patients with *ZNF750-wt* and *ZNF750-del*, and ESCC patients with *ZNF750-wt* and *ZNF750-Val* (*ZNF750-mut* + *ZNF750-del*). The horizontal axis is the survival time and the vertical axis is the percentage of survival cases in total cases. Every cross on the survival curves stands for censored data. Log-rank test *P*-value, Cox regression *P*-value, Hazard Ratio and 95% CI are displayed on the graph. **i** The mRNA of *ZNF750* was significantly decreased in ESCC tissue compared with paired normal tissue in 97 cases. Data represent the mean ± SD. All assays were performed in triplicate. Statistical analysis was performed with a two-side paired-*t* test. **p < 0.01, ***p < 0.0001.

In parallel, we profiled WGS on 508 pairs and RNA-sequencing on 97 pairs of ESCC, respectively. All FASTQ files are going to be uploaded to Genome Sequence Archive (GSA) in Beijing Institute of Genomics (BIG) Data Center, the accession number is HRA000021, that will be publicly accessible at http://bigd.big.ac.cn/gsa. Notably, *ZNF750* was identified as one of the most significantly mutated genes (SMGs, *q* value = 0) in this enlarged cohort, confirming that *ZNF750* may play critical roles in the development of ESCC. A total of 30 somatic mutations and 19 indels were detected in CDS region of *ZNF750* gene, that totally occurred in 48 out of 612 (7.84%) tumors (Fig. 1a). Of these mutations, 85.72% were inactivating mutations that include non-silent mutations (44.9%), indels (38.78%) and splice sites (2.04%), followed by missense mutations (14.29%) (Fig. S1a). Moreover, we observed recurrent mutations in noncoding regions of *ZNF750*, being mutated in 48 out of 508 (9.5%) tumors that have WGS data available. Of which, promoter mutations, that were found in 33 out of 508 patients (6.5%), were the most common somatic mutations (34.86% of total mutations), followed by intron (17.43% of total mutations) and untranslated regions (UTRs, 2.75% of total mutations) (Fig. S1b). Furthermore, analysis of focal copy number alterations (FCNA < 100 kb) across the 508 ESCC genomes revealed that *ZNF750* was present in a significantly deleted focal peak around 17q25.3 (*q* value = 8.72E–05) and *ZNF750* locus was to be deleted in 71 out of 508 tumors (14%, Fig. 1b, c), that was further validated by a real-time quantitative PCR (qRT-PCR) copy number assay (Fig. 1d, e).

Previously, we performed 104 pairs of WGS/WES from ESCC patients^4^, and then combined this cohort with the 508 pairs of WGS cohort (*n* = 612). To further estimate the associations between *ZNF750* mutations and/or deletions with clinicopathological features, rank sum and Chi-square (χ2) tests were applied in this enlarged cohort (*n* = 612). Although most features were not statistically significant, this analysis identified the following statistically significant patterns: (i) *ZNF750* mutations (*n* = 48 tumors) were significantly associated with late pathological stage (*P* = 0.000078), (ii) patients harboring *ZNF750* mutations exhibited more lymph node metastasis (*P* = 0.00011) and more distant metastasis (*P* = 0.001) compared with patients harboring no mutations in *ZNF750* (Table 1), and (iii) patients in subgroup of “*ZNF750-wt*” showed significantly better survival as compared to patients in subgroup of “mutated” (Kaplan–Meier analysis, *P* = 0.026; Cox regression, *P* = 0.029, hazard ratio (HR): 1.541, 95% confidence interval (CI): 1.044–2.261) (Fig. 1f). Strikingly, analysis of copy number alterations in 508 pairs of WGS revealed that *ZNF750* copy number loss (*n* = 71) was statistically associated with more lymph node metastasis (*P* = 0.046) compared with patients harboring no deletions in *ZNF750*. Subsequently, Kaplan–Meier’s analysis revealed that patients with *ZNF750* copy number loss developed more-frequent recurrence and had poorer survival (*P* = 0.048, Fig. 1g and Table S2). Cox’s proportional hazards regression analysis indicated that *ZNF750* deletion was an independent predictor for the ESCC prognosis. When *ZNF750* mutations and deletions were combined, the associations with late stage (*P* = 0.006), more lymph node metastasis (*P* = 0.000453), and poorer survival (*P* = 0.016) were highly significant (Fig. 1h and Table S3). Additionally, across 97 pairs of RNA-seq dataset, *ZNF750* expression level was significantly decreased in tumor samples in comparison with matched normal samples (Fig. 1i). Together, these findings suggest that there are clear associations between *ZNF750* mutations/deletions and clinical characteristics of Chinese ESCC patients, supporting the clinical potential of *ZNF750* mutations/deletions with its ability to estimate the metastasis and survival of patients with ESCC.

### Decreased *ZNF750* promoted tumor angiogenesis in ESCC

**Fig. 2:**
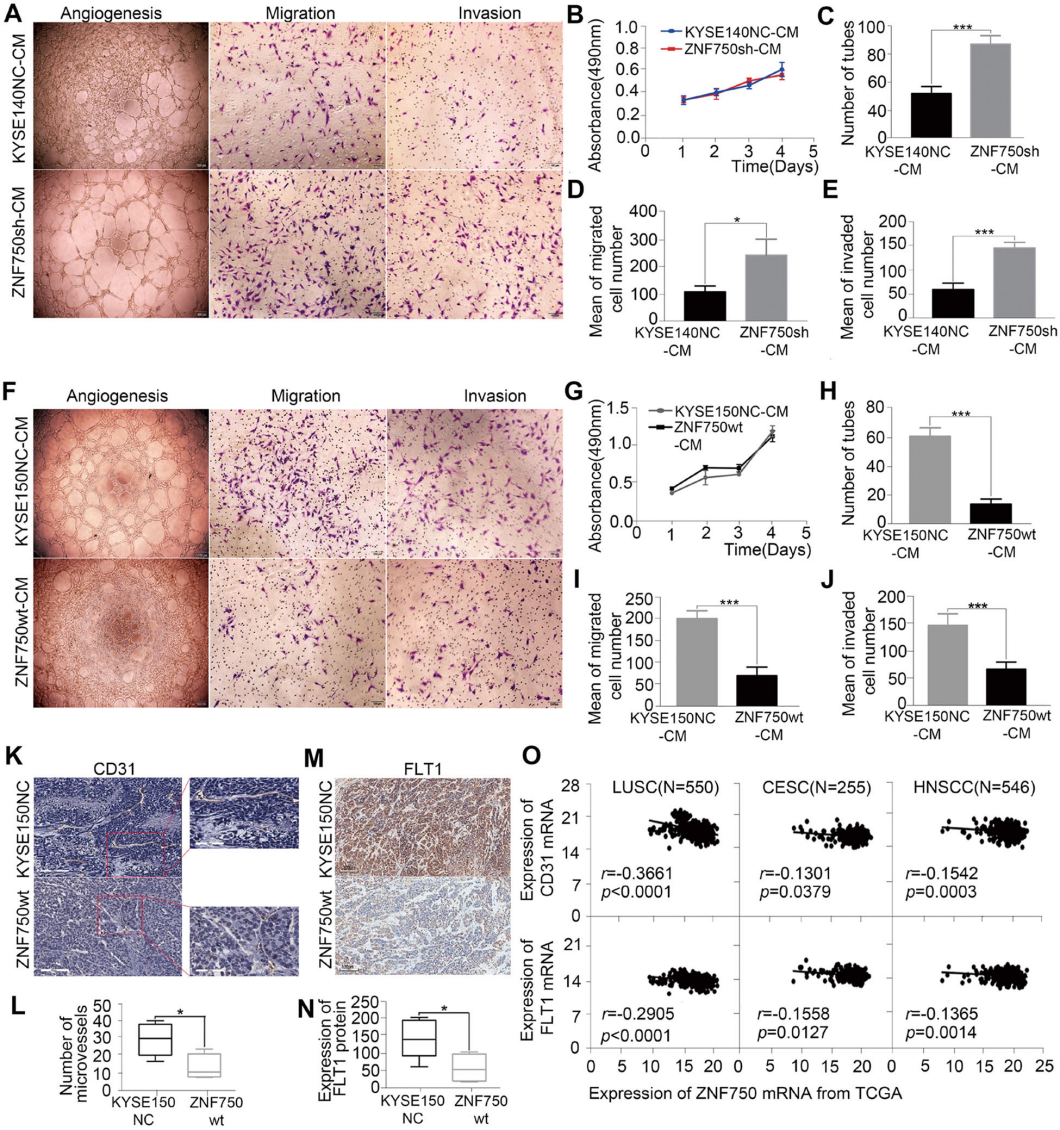
Decreased *ZNF750* promoted tumor angiogenesis in ESCC. **a** Representative images of conditioned medium of *ZNF750* knockdown cells promoted the tube formation (1st column), migration (2nd column), and invasion (3rd column) of HUVEC. **b**–**e** The conditioned medium of *ZNF750* knockdown cells promoted cell angiogenesis (**c**), migration (**d**) and cell invasion (**e**) but had no effect on cell proliferation (**b**) of HUVEC. **f** Representative images of conditioned medium of *ZNF750* overexpression cells inhibited the tube formation (1st column), migration (2nd column) and invasion (3rd column) of HUVEC. **g**–**j** The conditioned medium of *ZNF750* overexpression cells inhibited the tube formation (**h**), migration (**i**) and invasion (**j**) but had no effect on cell proliferation (**g**) of HUVEC. Data represent the mean ± SD. All assays were performed in triplicate. Statistical analysis was performed with a two-sided *t* test. **k**, **l** Representative immunostaining images show the microvessel density of tumor tissues from xenograft mouse injected with KYSE150NC more than that with *ZNF750-wt* cells (bar = 100 μm). **m**, **n** FLT1 were significantly reduced in *ZNF750-wt* animals. *P* values were obtained using non-paired *t* test. **o** Correlation analysis between ZNF750 and CD31,FLT1 in LUSC, CESC, HNSCC from TCGA. The horizontal axis is the expression of *ZNF750* mRNA and the vertical axis is the expression of *CD31* and *FLT1*. Statistical analysis was performed with pearson correlation. *p < 0.05, **p < 0.01, *** *p* < 0.001.

The pattern and significance of *ZNF750* genetic alterations and expression promote us to investigate the correlation of *ZNF750* with tumor metastasis in ESCC. Our previous study had showed *ZNF750* inhibited the proliferation and invasion of ESCC cells^4^. Angiogenesis plays a critical role in the growth and spread of cancer. We then explored the functional relevance of *ZNF750* with tumor angiogenesis in ESCC. We expressed *ZNF750*-sh in KYSE140 (Fig. S2a, b), and detected its effect on EA.hy926 cells, one human umbilical vein endothelial cell (HUVEC) line. We found that the proliferation rates of HUVEC were not significantly increased when treated with conditioned medium from KYSE140 cells with *ZNF750* knockdown. However, *ZNF750*-knockdown KYSE140 cell strongly promoted their ability of migration, invasion and tube formation in HUVEC cells (Fig. 2a–e). Similar results were observed in KYSE180 cells and KYSE450 cells (Fig. S2). In contrast, forced expression of *ZNF750-wt* in KYSE150 cells dramatically reverted the angiogenesis phenotypes of migration, invasion and tube formation of HUVEC cells in vitro **(**Fig. 2f–j). Consistent patterns were observed in HAEC, another human arterial endothelial cell line (Fig. S3).When transplanted *ZNF750* overexpression ESCC cells into subcutaneous, the tumor had a lower microvessel density compared with the control cells (Fig. 2k, l) and decreased expression of *CD31* and *FLT1*, two markers of angiogenesis phenotypes (Fig. 2m, n). Consistently, based on the TCGA data, a negative correlation between *ZNF750* and the expression of *CD31* and *FLT1* was observed in LUSC, CESC and HNSC, respectively (Fig. 2o). Collectively, these results indicate that *ZNF750* may be a critical suppressor of tumor angiogenesis to regulate metastasis of ESCC.

### *FOXC2* as a mediator of tumor angiogenesis induced by decreased *ZNF750*

**Fig. 3:**
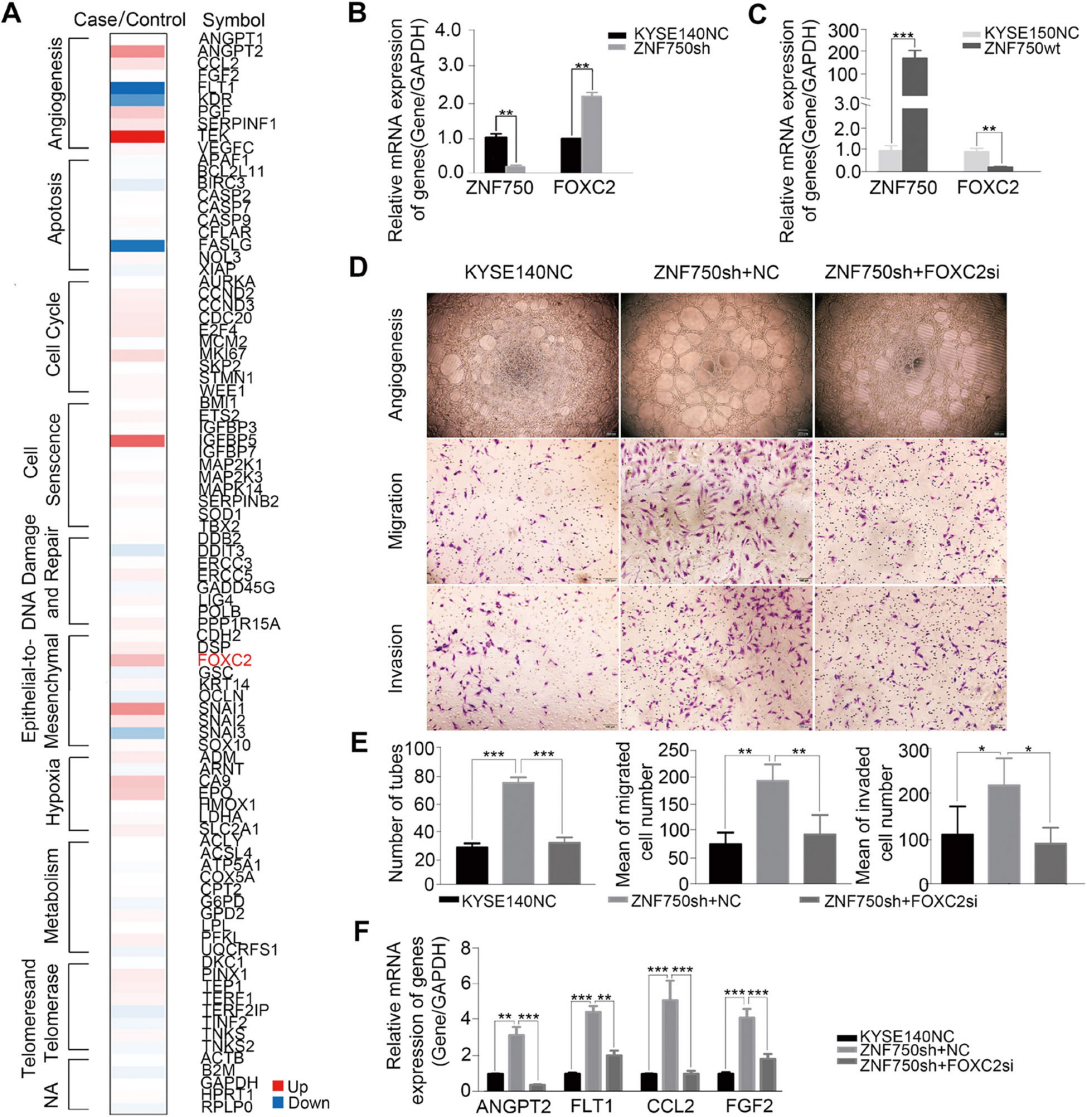
*FOXC2* as a mediator of tumor angiogenesis induced by decreased *ZNF750*. **a** PCR array in *ZNF750* knockdown cells using the kit of Cancer Pathway Finder PCR Array. **b**, **c** qRT-PCR showed the expression of *FOXC2* in *ZNF750* knockdown cells (**b**) and *ZNF750-wt* cells (**c**). GAPDH was performed as a loading control. Statistical analysis was performed with a two-sided *t* test. **d** Representative images of tube formation (**upper**), migration (**middle**) and invasion(**lower**) of HUVEC. **e** The conditioned medium from *FOXC2* knockdown in *ZNF750* knockdown cells reversed the effect on tube formation (**left**), migration (**middle**) and invasion(**right**) of HUVEC. **f** Angiogenic biomarkers *ANGPT2*, *FLT1*, *CCL2* and *FGF2* were detected by qRT-PCR in *ZNF750* knockdown cells and *FOXC2* knockdown in *ZNF750* knockdown cells. Statistical analysis was performed with one-way ANOVA. **p < 0.01, ***p < 0.0001.

Angiogenesis has been implicated as an essential component of tumor metastasis. To explore the potential molecular mechanism underlying decreased *ZNF750* induced angiogenesis and metastasis, we performed PCR array in *ZNF750* knockdown cells using the kit of Cancer Pathway Finder PCR Array and validated by qRT-PCR. The results showed that angiogenesis pathway was the top altered pathway, and *FOXC2*, a critical transcription factor regulating tumor angiogenesis and inducing epithelial-mesenchymal transition in various cancers, was significantly affected (Fig. 3a). qRT-PCR confirmed the increased expression of *FOXC2* in *ZNF750* knockdown cells and the decreased expression in *ZNF750*-wt forced expression KYSE150 cells (Fig. 3b, c). Furtherly, we found FOXC2 knockdown reversed the effect on angiogenesis in *ZNF750* knockdown cells. Importantly, knockdown FOXC2 in *ZNF750* knockdown KYSE140 cells decreased the ability of migration, invasion and tube formation in HUVEC cells treated with conditioned medium (CM) from *ZNF750* knockdown KYSE140 cells and KYSE450 cells, respectively (Fig. 3d, e and Fig. S4a, b). Similar patterns were observed in HAEC (Fig. S4c–f). As we know, FOXC2 has a paracrine effect on angiogenesis via regulation of its target gene angiopoietin 2 (Ang-2) and VEGFs^13^. We then detected the effect of ZNF750 and FOXC2 on Ang-2 and VEGFs, and found *ZNF750* knockdown induced up-regulation of Ang-2 and VEGFs in ESCC cells whereas downregulated FOXC2 reversed the phenomenon (Fig. 3f). Thus, FOXC2 may act as a mediator of tumor angiogenesis induced by *ZNF750* knockdown in ESCC.

### A long noncoding RNA (lncRNA) DANCR may be a downstream target of *ZNF750*

**Fig. 4:**
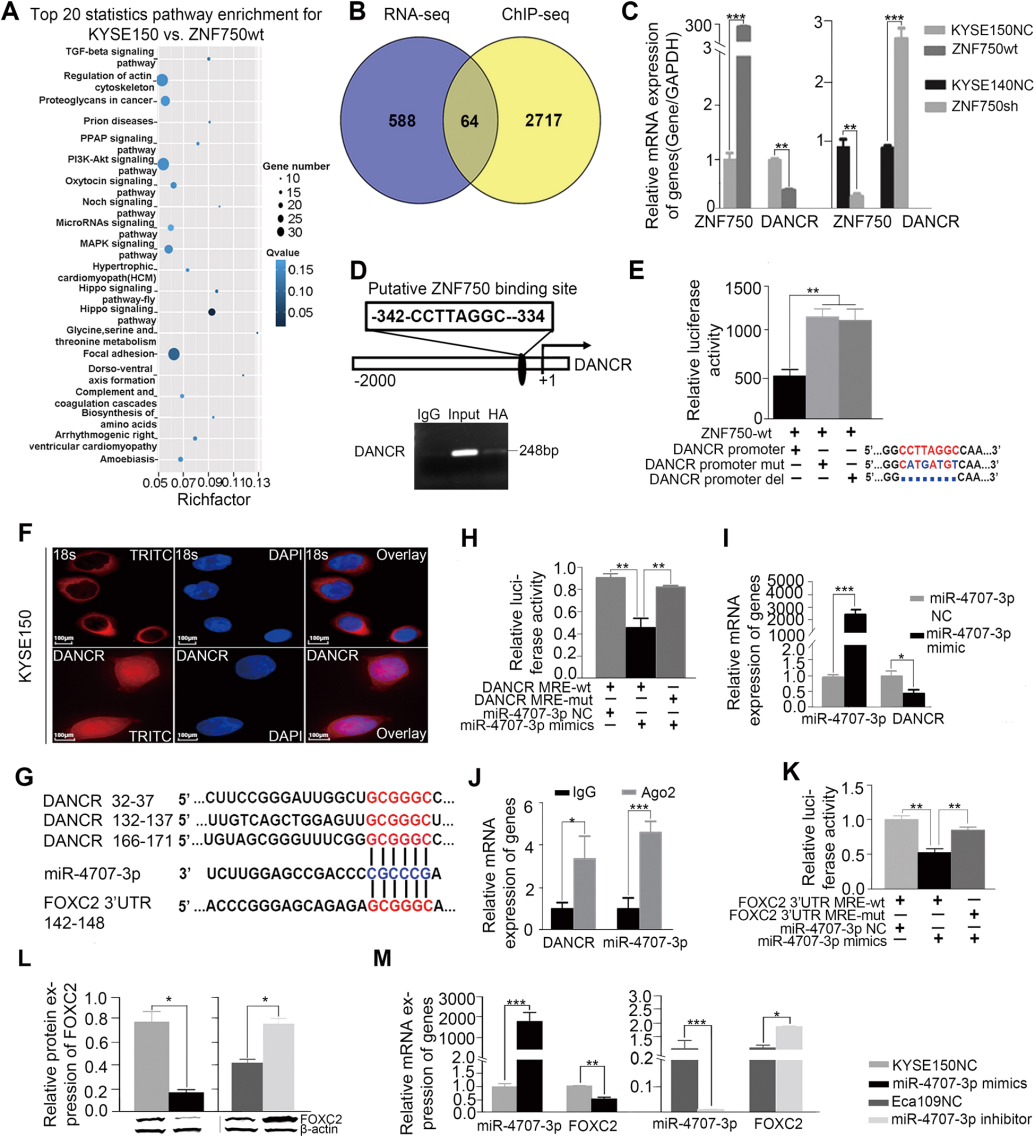
*DANCR* is a critical downstream target of *ZNF750* to regulate *FOXC2* expression in a ceRNA manner. **a** Pathway enrichment analysis displayed that the differentially expressed genes (Fisher-exact test, *p* < 0.05) were enriched in pathways such as PI3K/Akt signaling, Hippo signaling, regulation of actin cytoskeleton and proteoglycans in cancer. **b** 64 genes as potential direct targets of *ZNF750* occurring simultaneously in RNA-seq data and Boxer’s ChIP-sequencing data. **c** qRT-PCR showed the expression of *DANCR* in *ZNF750* knockdown cells (left) and *ZNF750-wt* cells (right). *GAPDH* was performed as a loading control. Statistical analysis was performed with a two-sided *t* test. **d** **Upper:** A putative ZNF750 binding site on the -342 to -334 region before transcriptional start site of *DANCR*. **Lower**: ChIP-PCR showed that ZNF750 directly bound to the promoter of *DANCR*. **e** Dual luciferase assay showed that *ZNF750* overexpression decreases the luciferase activity of *DANCR* reporter. This effect was abrogated when the ZNF750 binding site was mutated or deleted. *P* values were obtained using ANOVA. **f** *DANCR* localization in both cytoplasm and nuclear of KYSE150 cells as measured by RNA-FISH. DAPI labels the nucleus. Scale bars represent 100 μm. **g** The putative binding site of *DANCR* was found to be similar to the binding site of miR-4707-3p on 3’UTR of *FOXC2*. **h** Dual luciferase report assay showed *miR-4707-3p* mimics significantly reduced the luciferase activity of pSicheck2-*DANCR-wt* rather than pSicheck2-*DANCR-mut*. Statistical analysis was performed with a two-sided *t* test. (**i**) qRT-PCR showed the expression of *DANCR* in *miR-4707-3p inhibition* cells. **j** RIP showed *DANCR* and *miR-4707-3p* were presented as fold enrichment in Ago2 relative to IgG immunoprecipitates. Statistical analysis was performed with a two-sided *t* test. **k** Dual luciferase assay showed that *miR-4707-3p* mimics decreased the luciferase activity of *FOXC2*-3’UTR reporter rather than mutant *FOXC2*-3′UTR reporter. *P* values were obtained using ANOVA. **l**, **m** Augmentation of *miR-4707-3p* led to a decrease of *FOXC2* expression level whereas silence of *miR-4707-3p* caused an increase as measured by qRT-PCR (**l**) and western blot (**m**). Statistical analysis was performed with a two-sided *t* test. *p < 0.05, **p < 0.01,***p < 0.0001.

Although there was a putative binding site of ZNF750 on *FOXC2* promoter, Chromatin immunoprecipitation (ChIP) result showed ZNF750 could not bind its promoter region directly (Data not shown). To explore how ZNF750 contributes to regulation of *FOXC2* mRNA expression in ESCC, we performed RNA-sequencing to detect global alterations at mRNA level upon *ZNF750* overexpression in KYSE150 cells that have relatively low endogenous ZNF750 level. Pathway enrichment analysis displayed that the differentially expressed genes (Fisher-exact test, *P* < 0.05) were enriched in pathways such as PI3K/Akt signaling, Hippo signaling, regulation of actin cytoskeleton and proteoglycans in cancer (Fig. 4a). Next, we integrated our RNA-seq data with Boxer’s ChIP-sequencing data that explored the potential direct targets of *ZNF750* in keratinocytes^14^ and found 64 genes occurring simultaneously (Fig. 4b). Of these genes, 19 were identified containing putative ZNF750 binding sites within 2000 bp regions upstream of the transcription start site (TSS) (Fig. S5a). Six genes were downregulated upon *ZNF750* overexpression whereas up-regulated in *ZNF750* knockdown cells as demonstrated by qRT-PCR, that was consistent with RNA-seq data. Moreover, five genes (*HERC6*, *SPOCK2*, *CARD14*, *SEMA6D*, and *G6PD*) were positively correlated with ZNF750 expression level (Fig. S5b, c) whereas the lncRNA *DANCR* showed a negatively association with ZNF750 (Fig. 4c). In particular, *DANCR* has been implicated as an anti-differentiation ncRNA and was required for dedifferentiation of epidermal cells^15–17^. We then examined the direct interaction between ZNF750 and *DANCR via* ChIP and dual luciferase reporter assays in KYSE140 cells that harbor *ZNF750-wt* genotype. ChIP result demonstrated that ZNF750 bound to the promoter region from -342 to -334 upstream of TSS of *DANCR* (Fig. 4d). The dual luciferase assay showed that *ZNF750* overexpression significantly decreased the luciferase activity of *DANCR* promoter containing ZNF750 binding site, while the inhibitory effect was attenuated when the site was mutated or deleted (Fig. 4e), suggesting that ZNF750 directly bound to the promoter of *DANCR*, leading to an inhibition of *DANCR* expression.

### *DANCR* may be a critical downstream target of *ZNF750* to regulate *FOXC2* expression in a ceRNA manner

The predominant cytoplasm localization of *DANCR* in KYSE150 cells (Fig. 4f) suggests that *DANCR* tends to participate in post-transcriptional regulation by interacting with miRNAs. Interestingly, we found *DANCR* and the 3′-UTR of *FOXC2* mRNA had the similar binding sites (or called microRNA response element, MRE) of *miR-4707-3p* using miRTarBase and TargetScan (Fig. 4g). We co-transfected pSicheck2-*DANCR*-wt or -mut plasmid with miRNA-expression plasmids and subjected to luciferase report assay. Notably, we observed a 67% reduction of luciferase activity in *miR-4707-3p* expressed cells compared to that of empty vector control, meanwhile the suppression of luciferase activity was dramatically abolished by *DANCR*-mut expression(Fig. 4h), indicating that *miR-4707-3p* probably binds to the predicted MRE region of *DANCR*, thus downregulates *DANCR* expression (Fig. 4i). Moreover, RNA-Binding Protein Immunoprecipitation Assay (RIP) experiment using antibody against Ago2 in KYSE150 cell extracts showed that *DANCR* and *miR-4707-3p* were preferentially enriched in Ago2-containing miRNPs relative to the control IgG immunoprecipitates **(**Fig. 4j), indicating that *DANCR*, likely through interacting with *miR-4707-3p*, was present in Ago2-containing miRNPs. Similarly, as expect, *FOXC2* expression was negatively regulated by *miR-4707-3p* at the transcriptional level and post-transcriptional level. Silence of *miR-4707-3p* led to an increase of *FOXC2* mRNA and protein level in KYSE-150 cells whereas augmentation of *miR-4707-3p* caused a decrease of *FOXC2* in Eca109 cells (Fig. 4l, m). Dual luciferase reporter assay showed that *miR-4707-3p* inhibited the luciferase activity of *FOXC2*-3′UTR but had no significant influence on the luciferase activity of *FOXC2-*3′UTR*-*mut (Fig. 4k).

**Fig. 5:**
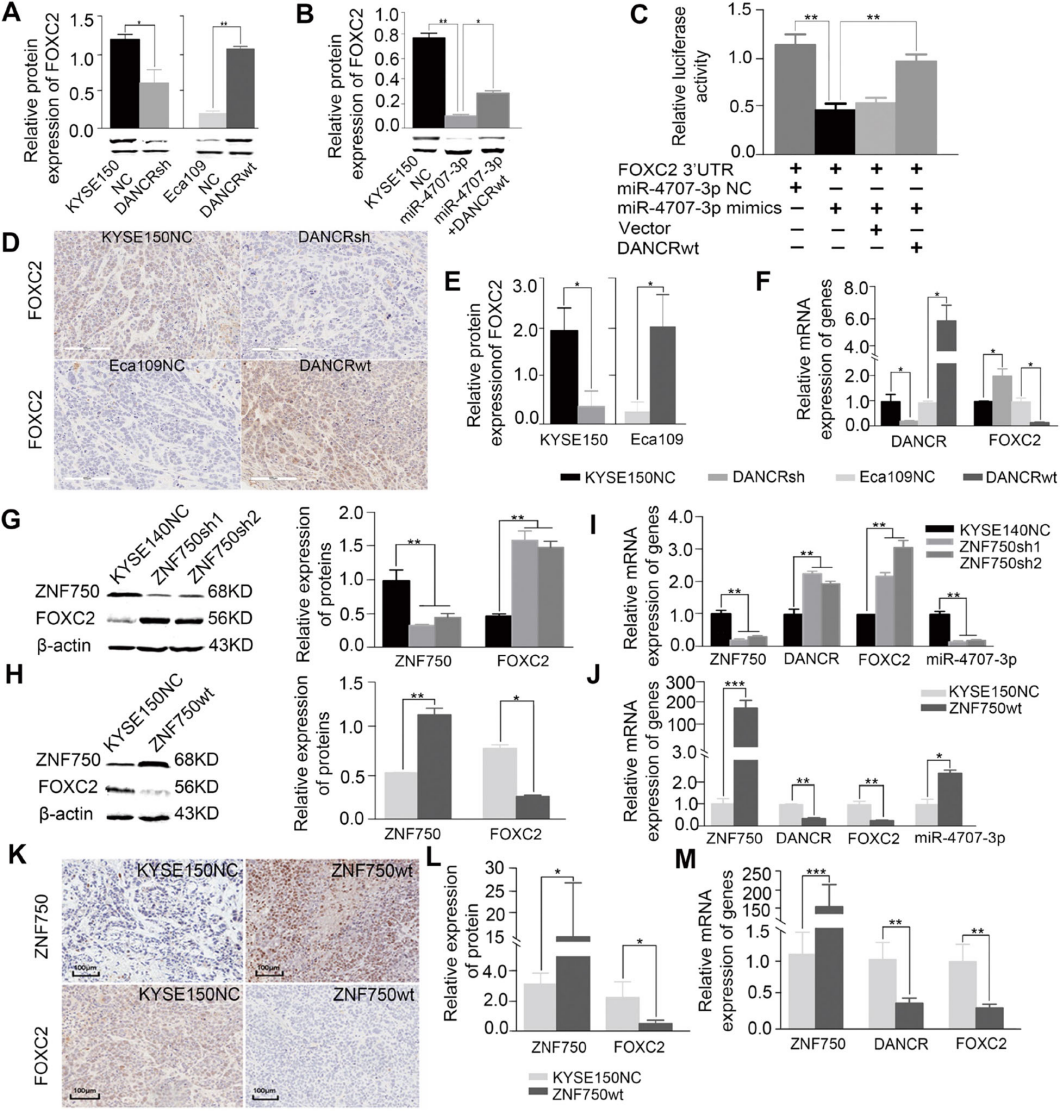
*ZNF750* may act as a tumor suppressor via *DANCR/miR-4707-3p/FOXC2* axis that works in a ceRNA manner in ESCC. **a, b** *DANCR* silence led to a decrease of FOXC2 protein level whereas augmentation of *DANCR* caused an increase in vitro (**a**), and the inhibition of FOXC2 protein was partially abolished by *DANCR-wt* overexpression in KYSE150-*miR-4707-3p* mimics cells (**b**). β-Actin was used as control. Statistical analysis was performed with a two-sided *t* test and one-way ANOVA. **c** Dual luciferase assay showed that the inhibitory effect of *miR-4707-3* on *FOXC2*-3’-UTR reporter was partially abolished by *DANCR-wt* overexpression. *P* values were obtained using ANOVA. **d**–**f** Immunohistochemistry (**d, e**) and qRT-PCR (**f**) showed the expression of FOXC2 in *DANCR* knockdown or overexpression xenograft mouse samples. GAPDH was used as loading control. Statistical analysis was performed with a two-sided *t* test. **g**–**j** Western blot (**g**, **h**) and qRT-PCR (**i, j**) showed the expression of *DANCR*, *miR-4707-3p*, FOXC2 in *ZNF750* knockdown and overexpression cells. β-Actin, *GAPDH* and *U6* were used as loading control respectively. Statistical analysis was performed with a two-sided *t* test. (**k**–**m**) Immunohistochemistry (**k**, **l**) and qRT-PCR (**m**) were used to detect the expression of *DANCR*, FOXC2 in *ZNF750* overexpression xenograft mouse samples. *GAPDH* was used as loading control. Statistical analysis was performed with a two-sided *t* test. *p < 0.05, **p < 0.01, *** *p* < 0.001.

Considering that both *DANCR* and *FOXC2* mRNA interact with *miR-4707-3p*, we then hypothesized that *DANCR* might function as a ceRNA for *FOXC2* to be modulated by *miR-4707-3p* in ESCC. To further explore this possibility, we detected the association between *DANCR* and FOXC2 expression and found that *DANCR* knockdown resulted in a decrease of FOXC2 expression whereas *DANCR* overexpression led to an increased expression of FOXC2 in vitro (Fig. 5a). Additionally, we also found FOXC2 expression was inhibited by *miR-4707-3p*, that was reversed when exogenous *DANCR* was expressed (Fig. 5b), suggesting that *DANCR* may compete with *FOXC2* to be targeted by *miR-4707-3p* in ESCC cells.

To further confirm the ceRNA regulatory mechanism among *DANCR*, *miR-4707-3p* and *FOXC2*, dual luciferase reporter assay was applied. As shown in Fig. 5c, the luciferase activity of *FOXC2*-3′UTR was inhibited by *miR-4707-3p*, this effect was attenuated by *DANCR-wt* expression. Moreover, the luciferase activity of *FOXC2*-3′UTR was impaired in *DANCR-wt* and *miR-4707-3p* mimics co-transfection group but not in *DANCR-mut* and *miR-4707-3p* mimics co-transfection group, indicating that the direct interaction between *DANCR-wt* and *miR4707-3p* is critical to abolish the negative regulation of *miR4707-3p* on *FOXC2*. We also found *DANCR* was positively with the expression of FOXC2 in xenograft mouse models at protein level (Fig. 5d, e) and mRNA level (Fig. 5f**)**. Together, this observation demonstrates that *DANCR* may act as a ceRNA to compete binding to *miR-4707-3p*, thus attenuates the inhibition of *miR-4707-3p* on *FOXC2* mRNA expression in ESCC cells.

### *ZNF750* may act as a tumor suppressor via *DANCR/miR-4707-3p/FOXC2* axis in ESCC

Given that the *DANCR/miR-4707-3p/FOXC2* axis in ESCC, we hypothesize ZNF750 may directly downregulate *DANCR* expression, strengthened the interaction of *miR-4707-3p* with *FOXC2*-3′UTR in a ceRNA manner, leading to degradation of *FOXC2* mRNA, thus playing tumor suppressive role in ESCC. To further validate this hypothesis, western blot and qPCR were used to detect the expression of *DANCR*, *miR-4707-3p*, FOXC2 in *ZNF750* knockdown or overexpression cells and xenograft mouse samples. Our results showed that *ZNF750* overexpression caused a remarkable decrease of *DANCR* and FOXC2 with a significant increase of miR-4707-3p whereas *ZNF750* knockdown led to a remarkable increase of *DANCR* and FOXC2 accompanied by a significant reduction of *miR-4707-3p* in vitro (Fig. 5g–j). The IHC results also showed FOXC2 was negatively with the expression of ZNF750 in xenograft mouse models at protein level (Fig. 5k, l) and mRNA level (Fig. 5m). In addition, we performed IHC in 508 ESCC tissues and paired normal esophagus tissues. As Fig. S7 shown, ZNF750 was markedly decreased in tumors compared to that of normal esophagus tissues, while FOXC2 was markedly increased in tumors compared to that of normal esophagus tissues. The results were consistent with the pattern observed in xenograft mouse models. Together, our results suggest that *ZNF750* plays a tumor suppressive role via *DANCR*/*miR-4707-3p*/*FOXC2* axis that works in a ceRNA manner in ESCC.

### *ZNF750* was negative correlated with *FOXC2* in multiple types of human squamous cell carcinoma

**Fig. 6:**
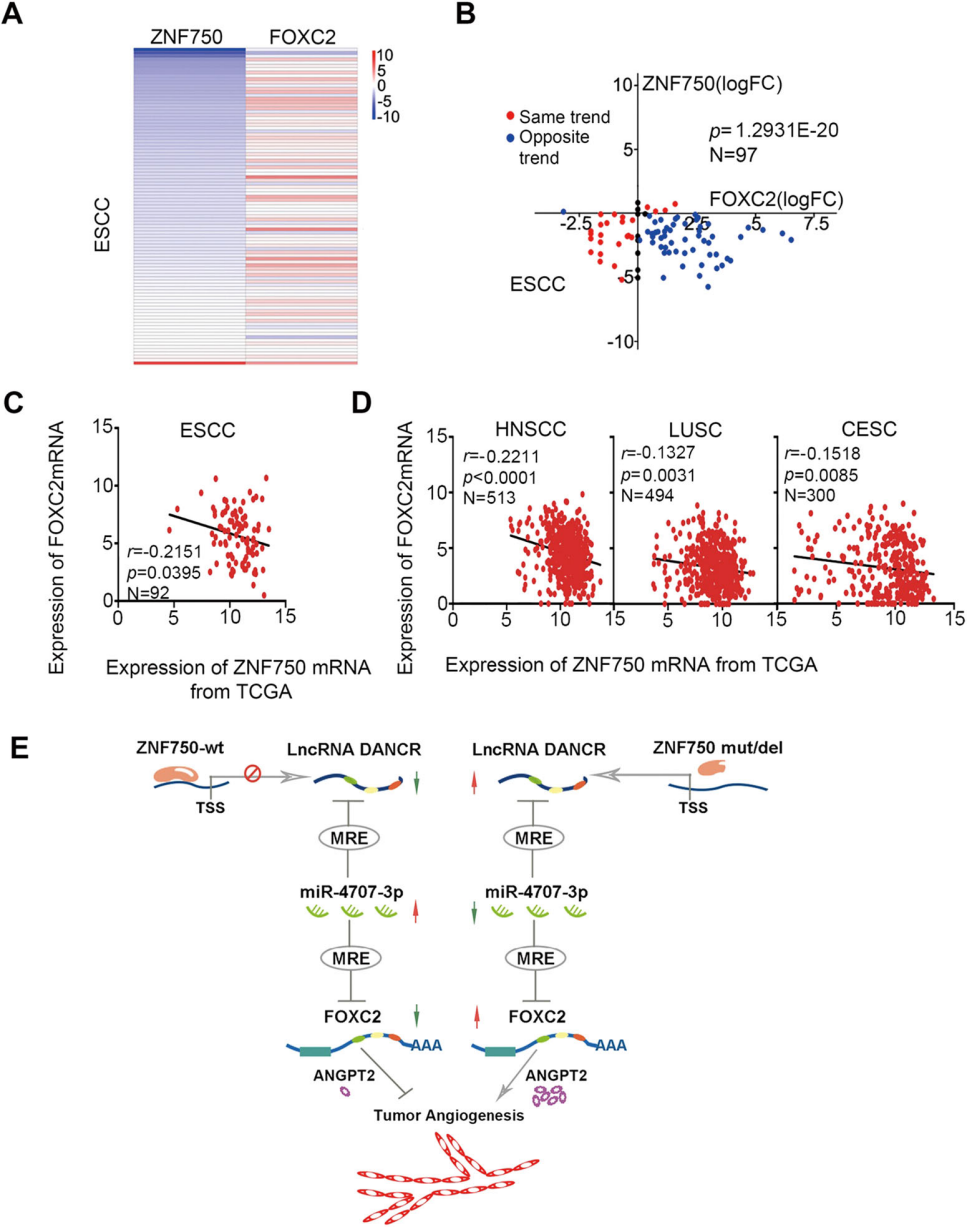
*ZNF750* is negative correlated with *FOXC2* in squamous cell carcinoma. **a** The log2 ratio heatmap of differences expression of *ZNF750* and *FOXC2* between ESCC tissues and paired normal tissues. Red represents up-regulation and blue represents down-regulation. **b** Correlation of differences expression between *ZNF750* mRNA and *FOXC2* mRNA. The horizontal axis is the log2 ratio of *FOXC2* and the vertical axis is the log2 ratio of *ZNF750*. Red dot showed the same differences expression trend between *ZNF750* and *FOXC2* mRNA. Blue dot showed the opposite trend between them. **c**–**e** Correlation analysis between *ZNF750* mRNA and *FOXC2* in 92 ESCC samples, 513 HNSC samples, 494 LUSC and 300 CESC samples from TCGA. The horizontal axis is the expression of *ZNF750* mRNA and the vertical axis is the mRNA expression of *FOXC2*. **f** Diagram showing how loss-function of *ZNF750* contributes to tumorigenesis of ESCC via *DANCR*/*miR-4707-3p*/*FOXC2* axis that works in a ceRNA manner.

Finally, we explored the correlation of ZNF750 with *FOXC2* expression level across various human squamous cell carcinomas. We analyzed their transcriptional level in ESCC based on our 95 pairs of RNA-seq data and found that *ZNF750* mRNA expression was negatively correlated with *FOXC2* mRNA level (*P* = 1.2931E-20, Fig. 6a, b). Additionally, we also conducted the correlation analysis of *ZNF750* with *FOXC2* transcriptional level in ESCC and other squamous cell carcinomas based on the TCGA database. Consistent with our paired ESCC samples, we found the negatively correlation of *ZNF750* mRNA with *FOXC2* mRNA in 92 ESCC cases (*r* = −0.2151, *P* = 0.0395), 513 cases of head and neck squamous cell carcinoma (*r* = −0.2211, *P* < 0.0001), 493 cases of squamous cell carcinoma of lung (*r* = −0.1327, *P* = 0.0031), and 300 cases of cervical squamous cell carcinoma (*r* = −0.1518, *P* = 0.0085), suggesting a possibility of ZNF750 deregulates *FOXC2* expression in human squamous cell carcinoma (Fig. 6c–e).These datas indicate a possibility of ZNF750 deregulates *FOXC2* expression in human squamous cell carcinoma. Together with our observations in vitro and in vivo, our findings suggest that *ZNF750*, a significantly mutated driver gene in ESCC, may regulate tumor angiogenesis via *DANCR*/*miR-4707-3p*/*FOXC2* axis that works in a ceRNA manner (Fig. 6f).

## Discussion

In this study, we profiled mutation and copy number alterations of *ZNF750* and uncovered its potential prognostic value for ESCC patients in an enlarged ESCC cohort. We applied genome sequencing approaches, in vitro and in vivo methods to reveal the tumor suppressor role of *ZNF750* and a possible novel mechanism underlying malignant phenotypes caused by loss-function of *ZNF750* in ESCC. Our data indicate that, for the first time, *ZNF750* copy number losses may be an attractive biomarker for risk metastasis and prognostication and *ZNF750* loss-of-function promotes tumorigenesis of ESCC *via DANCR*/*miR-4707-3p*/*FOXC2* axis in a ceRNA manner.

In view of previous whole-genome or -exome sequencing studies^4–7^, including our own, interrogated only a small number of samples, the global molecular portrait of ESCC remains incomplete. Profiling larger numbers of samples can help to identify cancer driver genes by providing the additional statistical power. Hence, our 612 WGS data could help us to understand the drivers of clinical phenotypes better. Consistent with previous reports^4,6,7,18,19^, *ZNF750* was identified as one of most SMGs in this enlarged cohort. Specially, more than 10% of 508 ESCC cases had focal copy number deletion, representing the most frequent molecular event reported on *ZNF750* gene. Together with several lines of functional evidences, *ZNF750* may work in a loss-of-function manner and its dysregulation may be crucial for tumor formation and progression in ESCC.

Clinically, we observed a significant correlation of patient *ZNF750* genotype with tumor phenotype. We found that individuals with mutations in *ZNF750* had a more later stage, more lymph node metastasis and much worse prognosis than individuals without mutations. And the results of *ZNF750* CNV were similar. Therefore, the correlation of *ZNF750* mutations and deletion with advanced disease stage and poor clinical outcome may explain the aggressive behavior of the *ZNF750* mutated ESCC. Although previous studies reported several potential biomarkers associated with clinical features especially patients’ outcome^5,7^, the statistics power of these biomarkers became weaker in further larger validate cohort, resulting in the clinical significance of genetic abnormalities of ESCC remains poorly defined and no reliable molecular markers for diagnosing or predicting patient outcomes have been identified to date^20^. Given that the greatly increased statistical power with our 612-case population, our finding highlights the clinical potential of *ZNF750* for Chinese ESCC patients and warrant further clinical investigation through prospective randomized clinical trials to confirm the application.

Currently, the treatment of ESCC relies on surgery, chemotherapy, radiotherapy, or combinations of these^2,21^, but limited on effective molecularly targeted therapies that may attribute to the precise molecular events underlining ESCC formation and metastasis remain only partially understood. In this study, we found that loss-function of *ZNF750* significant promoted tumor angiogenesis, which indicates anti-angiogenesis might be an efficient method in the inhibition of the growth and metastasis of ESCC with *ZNF750* mutation or deletion. Furtherly, we found the effect of ZNF750 is via *DANCR/miR-4707-3p/FOXC2* axis in a ceRNA manner in ESCC. Recent studies have implicated that *DANCR* might act as an oncogenic lncRNA in tumor progression such as hepatocellular carcinoma^18,22^, prostate cancer^23^, colorectal cancer^24^, glioma^25^, gastric cancer^26^, osteosarcoma^27^, lung adenocarcinoma^28^. In agreement with previous reports on various types of human cancers, *DANCR* may also act as an oncogene in ESCC progression, supporting that *DANCR* may offer a potential therapeutic target for those ESCC patients harboring mutations of *ZNF750*.

It is well known that lncRNAs can affect cellular behavior by diverse mechanisms^29–31^, for example, direct interaction with microRNAs. In this study, the interaction between *DANCR* and *miR-4707-3p* was found to regulate *FOXC2* expression in a ceRNA manner. *ZNF750* loss-of-function led to increased *DANCR* expression level that competed to bind to *miR-4707-3p*, attenuated the degradation of oncogene *FOXC2* by *miR-4707-3p*, thus promoted angiogenesis in ESCC cells. *MiR-4707-3p* was previously rarely reported in human cancers. Functionally, knockdown of *miR-4707-3p* promoted tumor angiogenesis probably by releasing the oncogenic role of *FOXC2*. *FOXC2* has been implicated as an oncogene promoting tumor invasion and metastasis in various of human cancers, for example, it mediated epithelial-mesenchymal transition^32^, multidrug resistance^33^, or regulator of vascular development in basal-like breast cancer, non-small cell lung cancer^34^, colon cancer^35^. *FOXC2* also has a paracrine effect on angiogenesis via regulation of its’ target gene Angiopoietin 2 (Ang-2) and VEGFs^13^. Our functional results also support the oncogenic roles of *FOXC2* in ESCC. Hence, besides the *DANCR*, our study provides evidence to support additional potential therapeutic target, *FOXC2*, for those ESCC patients with *ZNF750* mutations.

In summary, our study, for the first time, explore a novel *DANCR/miR-4707-3p/FOXC2* regulatory pathway through which *ZNF750*, a significant mutated driver gene, may deregulate angiogenesis involving in ESCC tumorigenesis and progress. We also highlight its potential to impact clinical outcomes of patients and offer potential therapeutic targets for ESCC treatment. Further efforts are required to exploit this information to develop prognostic method and to identify therapeutic targets that could be used to treat biomarker-selected groups of patients with ESCC.

## Supplementary information

Supplementary Figure and Table legends

Figure S1

Figure S2

Figure S3

Figure S4

Figure S5

Figure S6

Figure S7

Table S1

Table S2

Table S3
