# Supplementary material for: Decreased ZNF750 promotes angiogenesis in a paracrine manner via activating DANCR/miR-4707-3p/FOXC2 axis in esophageal squamous cell carcinoma
Source: Cell Death Dis. 2020 Apr 27;11(4):296. doi: 10.1038/s41419-020-2492-2 (PMC7186230; doi:10.1038/s41419-020-2492-2)
Supplement: Supplementary file 1 — Supplementary Figure and Table legends [file 41419_2020_2492_MOESM1_ESM.docx]

**Figure S1. The mutation frequency of *ZNF750* in coding and non-coding regions.**

1. In coding region of *ZNF750*, 85.72% were inactivating mutations that includes nonsense mutations (44.9%), indels (38.78%) and splice sites (2.04%), followed by missense mutations (14.29%). **(B)** In noncoding region of *ZNF750*, promoter mutations, that were found in 33 out of 508 patients (6.5%), were the most common somatic mutations (34.86% of total mutations), followed by intron (17.43% of total mutations) and untranslated regions (UTRs, 2.75% of total mutations).

**Figure S2. Decreased *ZNF750* promoted tumor angiogenesis in KYSE180 and KYSE450 cells.**

1. The protein expression pattern of ZNF750 in various of ESCC cell lines were detected by western blot. **(B-D)** Knockdown efficiency of ZNF750 in KYSE140 cells (**B**), KYSE180 cells (**C**) and KYSE450 cells (**D**) were shown by western blot. **(E)** Overexpression efficiency of ZNF750 in KYSE150 cells were shown by western blot. **(F and K)** Representative images of the tube formation (upper), migration (middle), and invasion (lower) of HUVEC. **(G-J and L-O)** Conditioned medium of KYSE180-*ZNF750*／KYSE450-*ZNF750* knockdown cells promoted cell angiogenesis, migration and cell invasion but had no effect on cell proliferation of HUVEC. Data represent the mean ± SD. All assays were performed in triplicate. Statistical analysis was performed with a two-sided t test. **p* < 0.05, ***p* < 0.01, ****p* < 0.0001.

**Figure S3. Decreased *ZNF750* promoted tumor angiogenesis in HAEC.**

**(A and F)** Representative images of the tube formation (1^st^ column), migration (2^nd^ column), and invasion (3^rd^ column) of HAEC cultured in the conditioned medium of *ZNF750* knockdown KYSE140(**A**), KYSE180 and KYSE450(**F**) cell. **(B, J and K)** The conditioned medium of *ZNF750* knockdown cells had no effect on HAEC proliferation. **(C-E and G-I)** The conditioned medium of *ZNF750* knockdown cells promoted the tube formation, migration and invasion of HAEC. **(L)** Representative images of the tube formation (1^st^ column), migration (2^nd^ column), and invasion (3^rd^ column) of HAEC cultured in the conditioned medium of *ZNF750* overexpression KYSE150(**L**) cell. **(M-P)** The conditioned medium of *ZNF750* overexpression cells inhibited the tube formation, migration and invasion but had no effect on cell proliferation of HAEC. Data represent the mean ± SD. All assays were performed in triplicate. Statistical analysis was performed with a two-sided t test. **p* < 0.05, ***p* < 0.01, ****p* < 0.0001.

**Figure S4. Decreased FOXC2 inhibited tumor angiogenesis in ESCC.**

(**A**) Representative images of the tube formation (upper), migration (middle), and invasion (lower) of HUVEC. (**B**) Knockdown FOXC2 in *ZNF750* knockdown cells decreased the ability of migration, invasion and tube formation in HUVEC cells treated with conditioned medium (CM) from *ZNF750* knockdown KYSE450 cells. (**C and E**) Representative images of the tube formation (upper), migration (middle), and invasion (lower) of HAEC. (**D and F**) Knockdown FOXC2 in *ZNF750* knockdown cells decreased the ability of migration, invasion and tube formation in HAEC cells treated with conditioned medium (CM) from *ZNF750* knockdown KYSE140 and KYSE450 cells, respectively. *P* values were obtained using ANOVA. **p* < 0.05, ***p* < 0.01, ****p* < 0.0001.

**Figure S5. *DANCR* may be a critical downstream target of *ZNF750*.**

**(A)**19 were identified containing putative ZNF750 binding sites within 2000 bp regions upstream of the transcription start site (TSS). **(B-C)** Six genes (*HERC6*, *SPOCK2*, *CARD14*, *SEMA6D*, *G6PD* and *DANCR*) were down-regulated upon *ZNF750* overexpression (**B**) whereas up-regulated in *ZNF750* knockdown cells (**C**) as demonstrated by qRT-PCR.

**Figure S6. ZNF750 inhibited the expression of FOXC2 and ANGPT2 in xenograft mouse.**

Immunohistochemistry (**A-B**) and qRT-PCR (**C**) were used to detect the expression of *DANCR*, FOXC2 and ANGPT2 in *ZNF750* overexpression xenograft mouse samples. *GAPDH* was used as loading control. Statistical analysis was performed with a two-sided t test. *p < 0.05, **p < 0.01, *** p <0.001.

**Figure S7. Expression of ZNF750 and FOXC2 in 508 ESCC tissues.**

**(A)** Represent images display strongly nuclear positivity in normal esophagus tissues. (**B**) Expression of ZNF750 was markedly increased in normal esophagus tissues compared to that of ESCC tissue based on judgment of IHC staining intensity. (**C**) Represent images display strongly nuclear and cytoplasm positivity in ESCC tissues. (**D**) Expression of FOXC2 was markedly decreased in normal esophagus tissues compared to that of ESCC tissue based on judgment of IHC staining intensity. Statistical analysis was performed with a rank sum t test. *p < 0.05, **p < 0.01, *** p <0.001.
