# Supplementary figures and images for: Decreased ZNF750 promotes angiogenesis in a paracrine manner via activating DANCR/miR-4707-3p/FOXC2 axis in esophageal squamous cell carcinoma
Source: Cell Death Dis. 2020 Apr 27;11(4):296. doi: 10.1038/s41419-020-2492-2 (PMC7186230; doi:10.1038/s41419-020-2492-2)

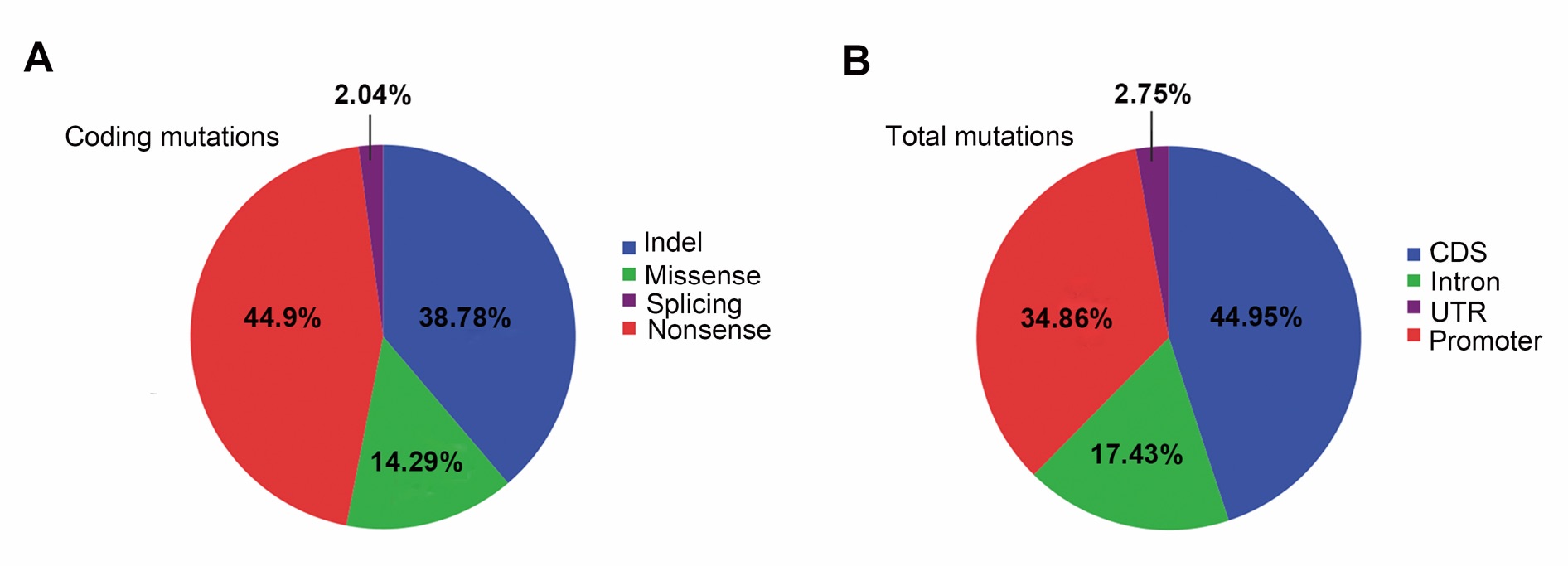

Supplement: Supplementary file 2 — Figure S1 [file 41419_2020_2492_MOESM2_ESM.jpg]

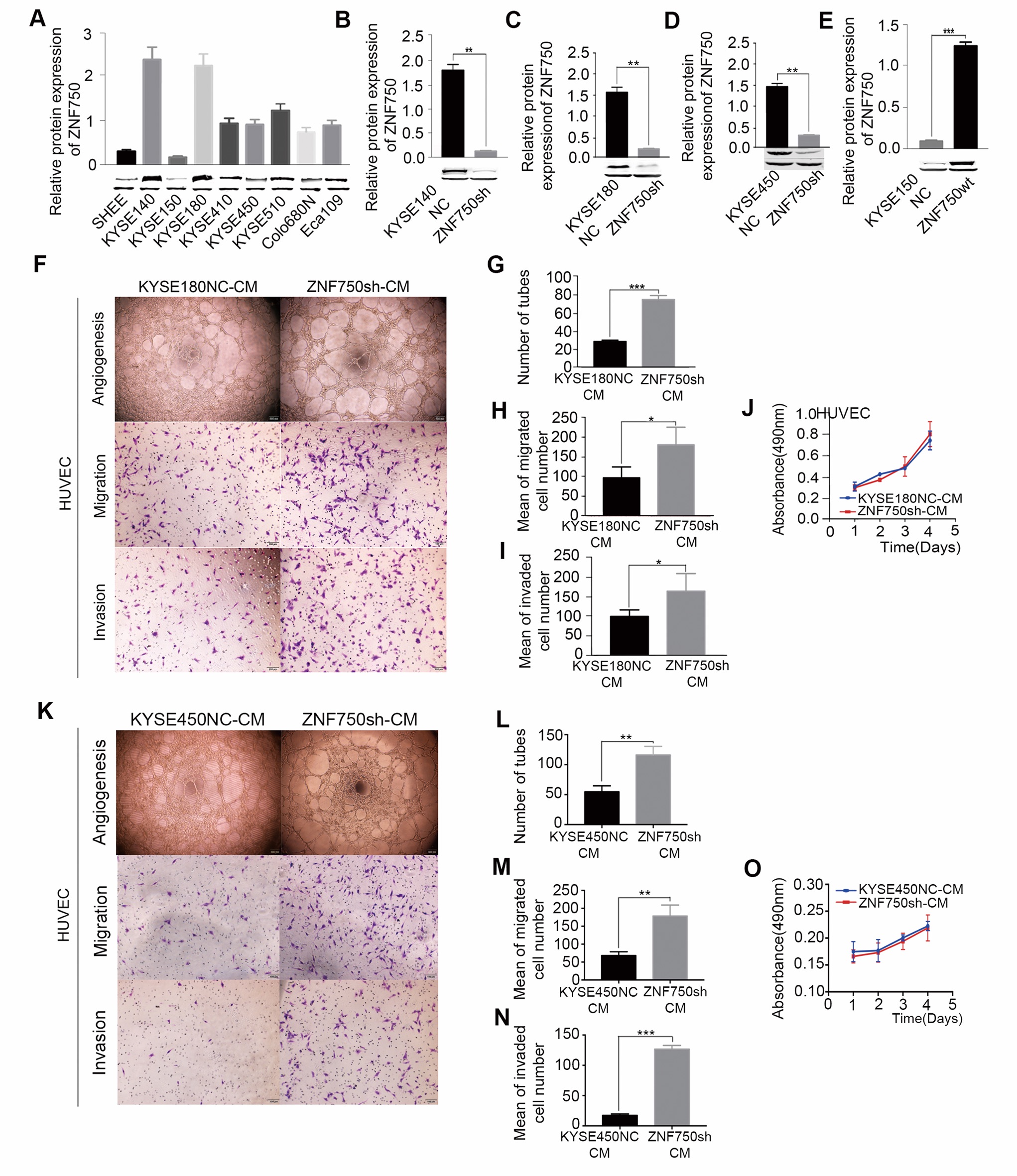

Supplement: Supplementary file 3 — Figure S2 [file 41419_2020_2492_MOESM3_ESM.jpg]

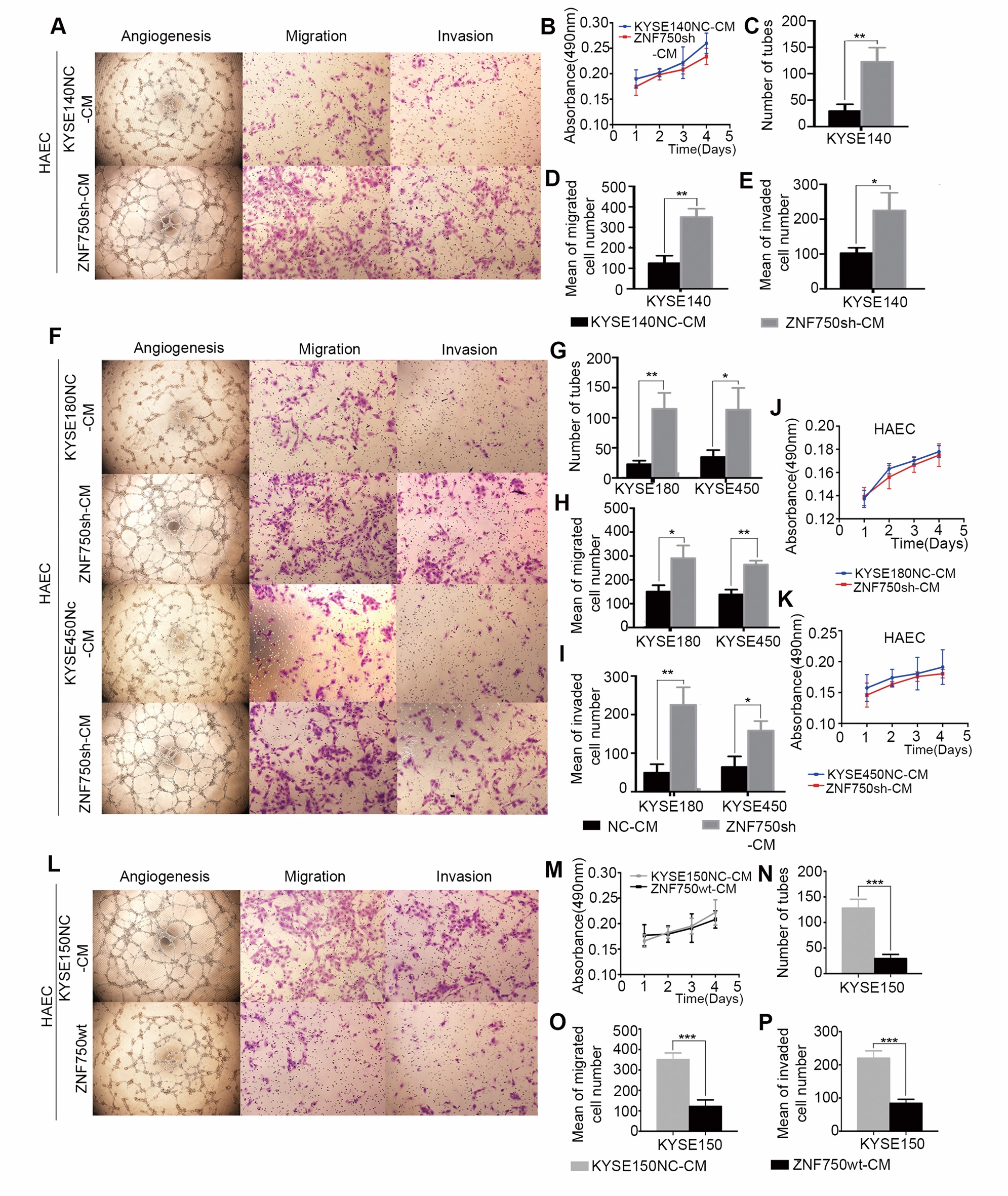

Supplement: Supplementary file 4 — Figure S3 [file 41419_2020_2492_MOESM4_ESM.jpg]

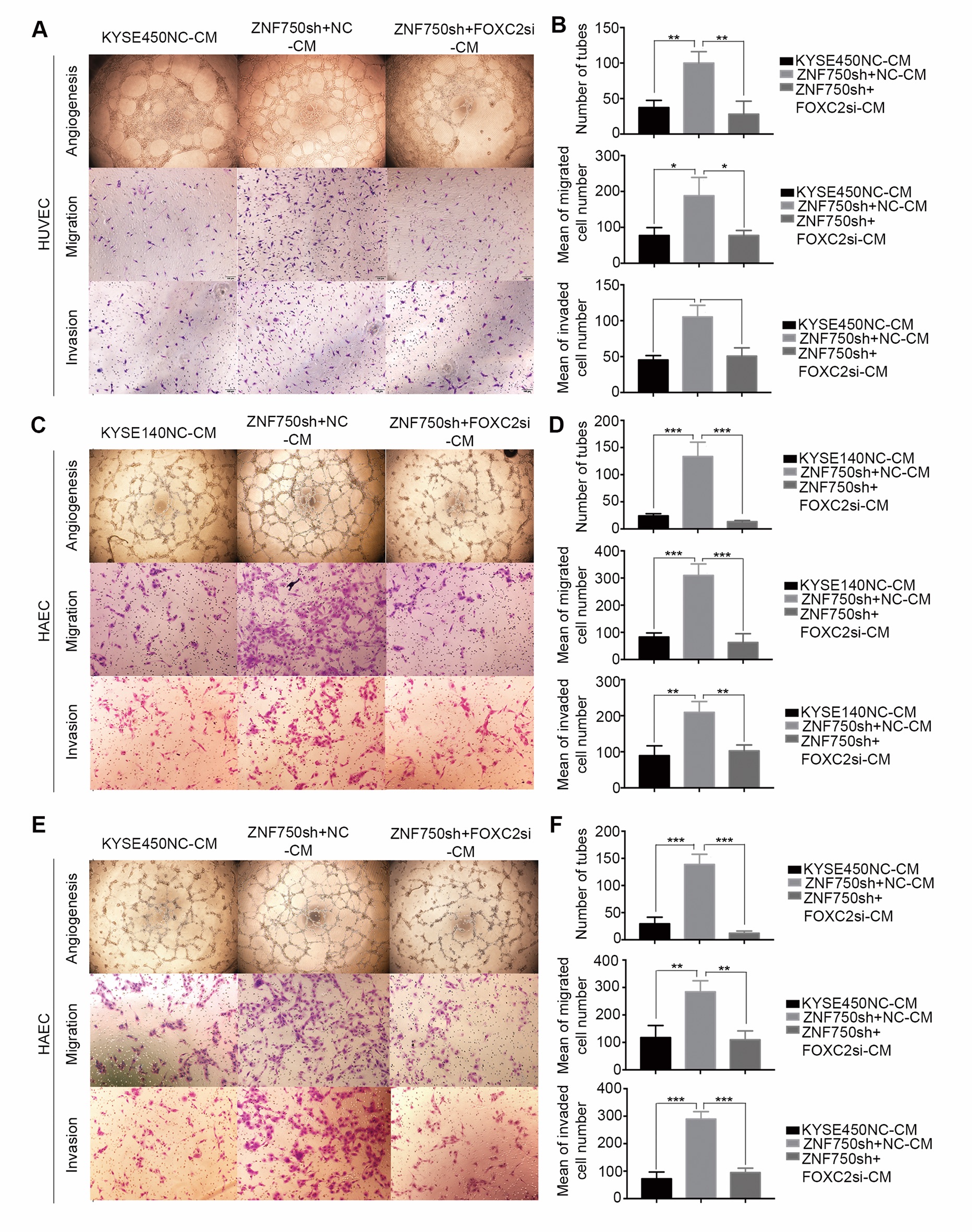

Supplement: Supplementary file 5 — Figure S4 [file 41419_2020_2492_MOESM5_ESM.jpg]

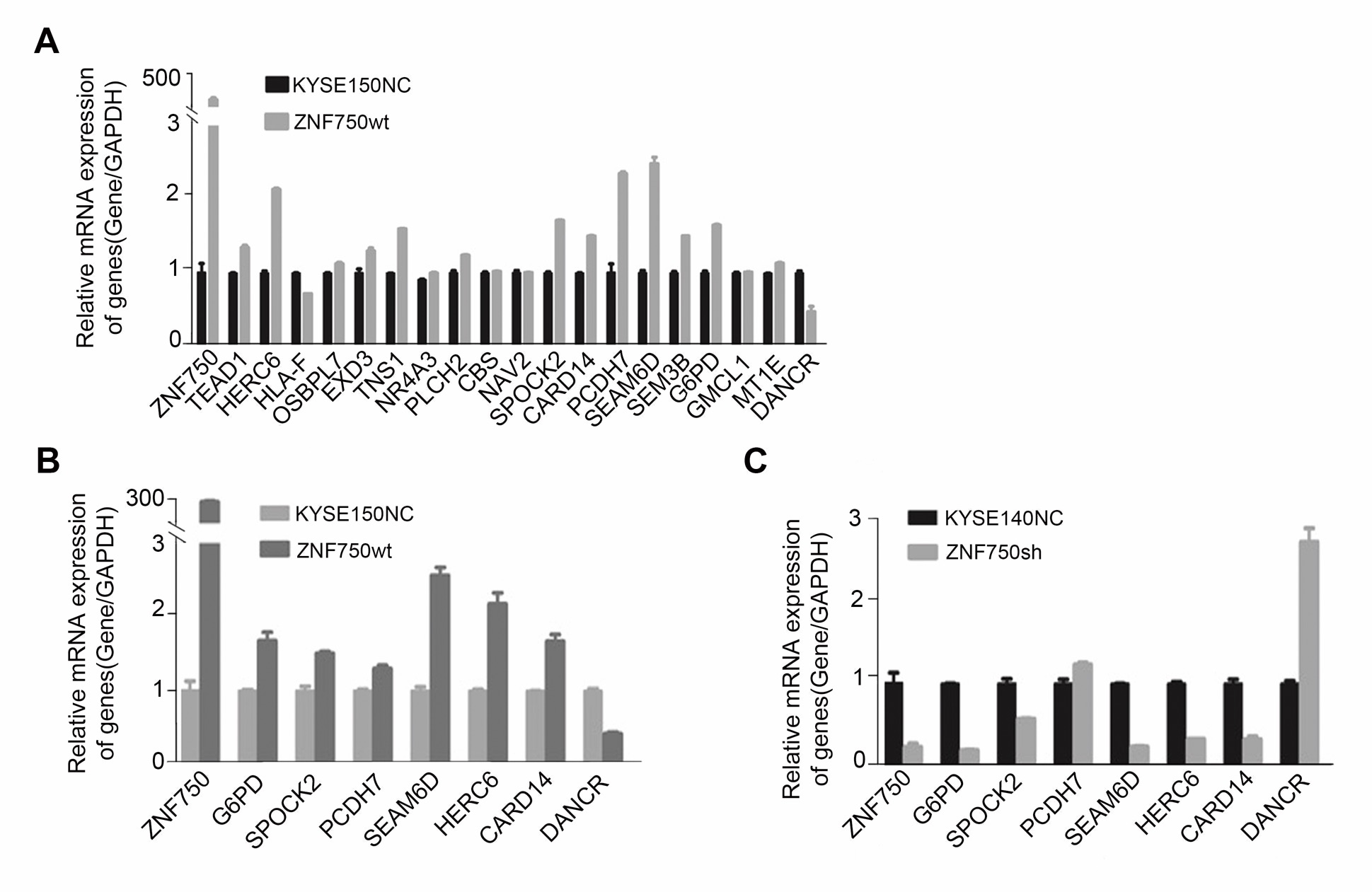

Supplement: Supplementary file 6 — Figure S5 [file 41419_2020_2492_MOESM6_ESM.jpg]

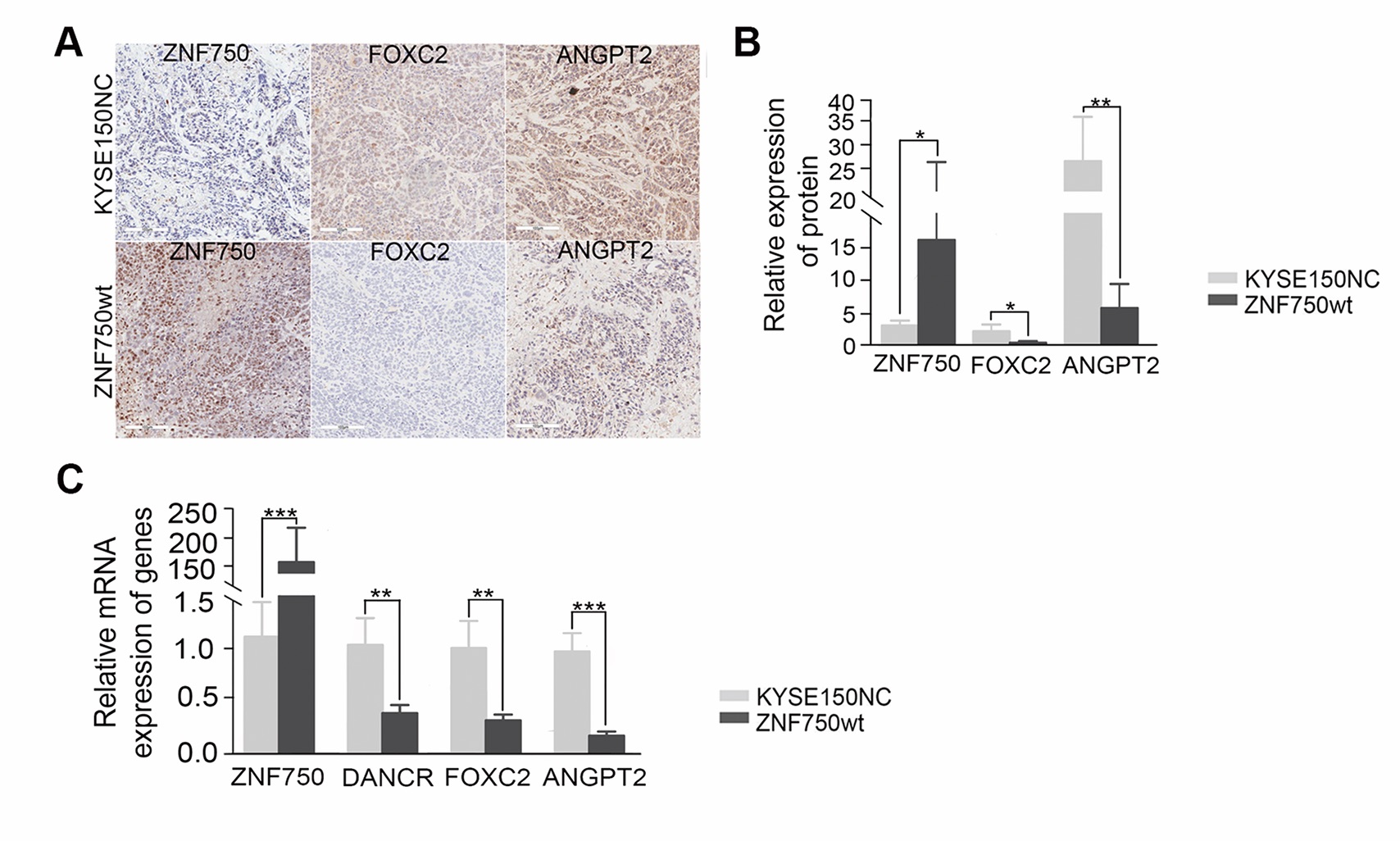

Supplement: Supplementary file 7 — Figure S6 [file 41419_2020_2492_MOESM7_ESM.jpg]

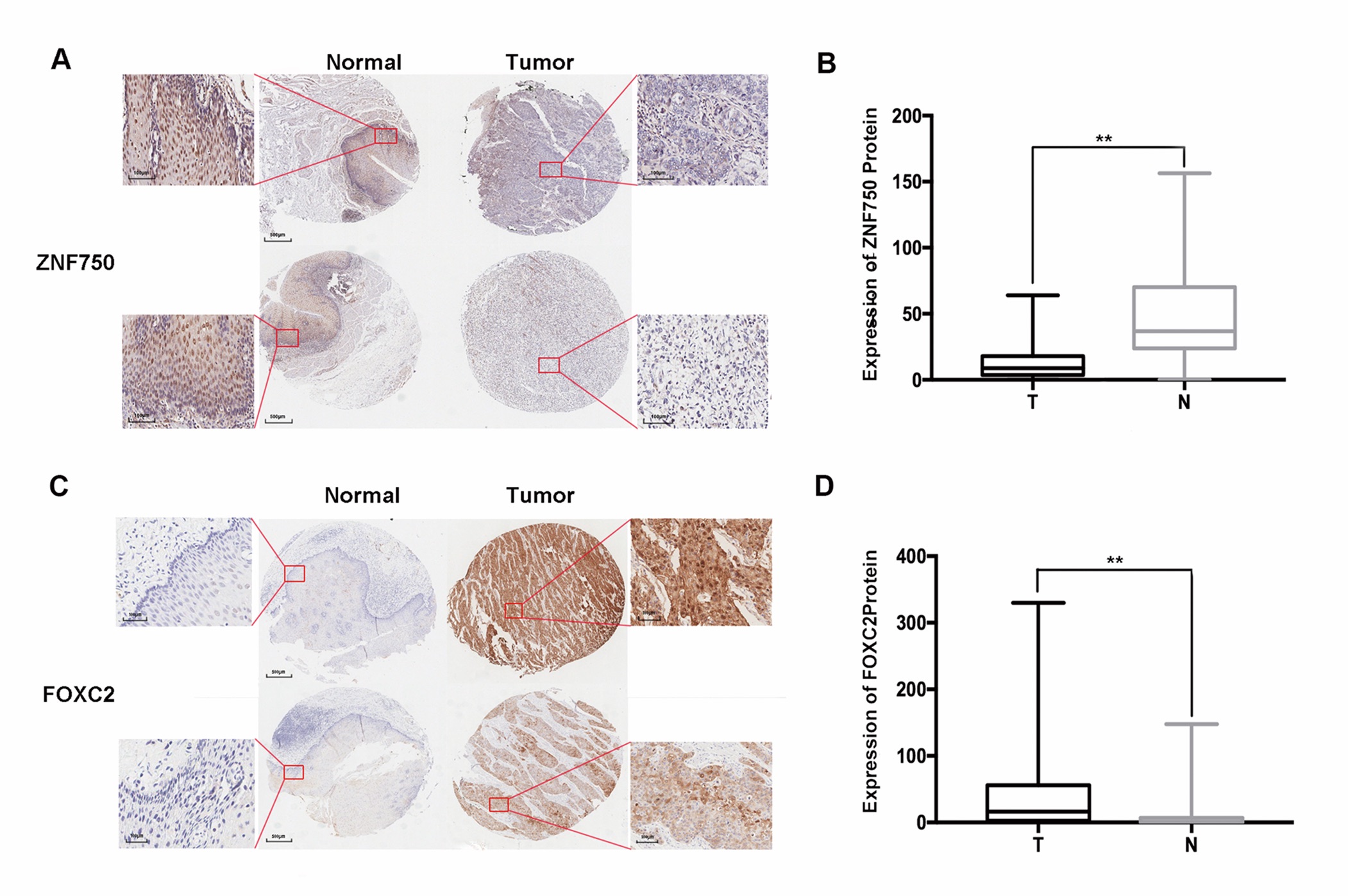

Supplement: Supplementary file 8 — Figure S7 [file 41419_2020_2492_MOESM8_ESM.jpg]
